# The Effect of Radiation on the Gut Bacteriome of *Aedes albopictus*

**DOI:** 10.3389/fmicb.2021.671699

**Published:** 2021-07-08

**Authors:** Dongjing Zhang, Shi Chen, Adly M. M. Abd-Alla, Kostas Bourtzis

**Affiliations:** ^1^Insect Pest Control Laboratory, Joint FAO/IAEA Centre of Nuclear Techniques in Food and Agriculture, Vienna, Austria; ^2^Key Laboratory of Tropical Disease Control of the Ministry of Education, Sun Yat-sen University–Michigan State University Joint Center of Vector Control for Tropical Diseases, Zhongshan School of Medicine, Sun Yat-sen University, Guangzhou, China; ^3^Chinese Atomic Energy Agency Center of Excellence on Nuclear Technology Applications for Insect Control, Sun Yat-sen University, Guangzhou, China; ^4^Institute of Biological Control, Fujian Agricultural and Forestry University, Fuzhou, China

**Keywords:** gut bacteriome, *Elizabethkingia*, *Aeromonas*, 16S *rRNA* gene, *Aedes albopictus*

## Abstract

The sterile insect technique (SIT) has been developed as a component of area-wide integrated pest management approaches to control the populations of *Aedes albopictus*, a mosquito vector capable of transmission of dengue, Zika and chikungunya viruses. One of the key factors for the success of SIT is the requirement of high biological quality sterile males, which upon their release would be able to compete with wild males for matings with wild females in the field. In insects, gut bacteriome have played a catalytic role during evolution significantly affecting several aspects of their biology and ecology. Given the importance of gut-associated bacterial species for the overall ecological fitness and biological quality of their hosts, it is of interest to understand the effects of radiation on the gut-associated bacteriome of *Ae. albopictus*. In this study, the effect of radiation on the composition and density levels of the gut-associated bacterial species at the pupal stage as well as at 1- and 4-day-old males and females was studied using 16S *rRNA* gene-based next generation sequencing (NGS) and quantitative PCR (qPCR) approaches. Age, diet, sex, and radiation were shown to affect the gut-associated bacterial communities, with age having the highest impact triggering significant changes on bacterial diversity and clustering among pupae, 1- and 4-day-old adult samples. qPCR analysis revealed that the relative density levels of *Aeromonas* are higher in male samples compared to all other samples and that the irradiation triggers an increase in the density levels of both *Aeromonas* and *Elizabethkingia* in the mosquito gut at specific stages. Our results suggest that *Aeromonas* could potentially be used as probiotics to enhance protandry and sex separation in support of SIT applications against *Ae. albopictus*, while the functional role of *Elizabethkingia* in respect to oxidative stress and damage in irradiated mosquitoes needs further investigation.

## Introduction

Mosquito-borne diseases including malaria, dengue, Zika, chikungunya, West Nile, yellow fever, and Japanese encephalitis cause severe burden on public health worldwide (Tolle, 2009). The sterile insect technique (SIT) is a species-specific and environment-friendly approach, which has been proposed and currently tested for the control of mosquito populations and mosquito-borne diseases (Lees et al., 2015; Bourtzis et al., 2016; Bouyer et al., 2020; WHO and IAEA, 2020). An important factor in SIT applications is male mating competitiveness, which is the ability of the mass-reared and radiation-sterilized males to compete with wild males for mating with wild females (Knipling, 1955; Dyck et al., 2020). Several studies have shown that mass-rearing, radiation, handling, marking, and release processes may affect male mating competitiveness and hence, the suppression efficiency of the SIT application (Helinski and Knols, 2008; Ami et al., 2010; Bellini et al., 2013; Maïga et al., 2014; Yamada et al., 2014; Cai et al., 2018; Diallo et al., 2019; Bouyer and Vreysen, 2020; Dyck et al., 2020).

Insects are known to have established diverse symbiotic associations with microbial species (particularly of bacterial origin), which have played a catalytic role during evolution significantly affecting several aspects of their biology and ecology (Dillon and Dillon, 2004; Bourtzis and Miller, 2009; Zchori-Fein and Bourtzis, 2011; Engel and Moran, 2013). During the recent years, a number of studies have characterized the gut-associated microbiota of several insect species, including pests and disease vectors, in an attempt to determine the role of microbial species (mainly bacteria) in host physiology, and also to potentially exploit them as a tool for pest and disease control (Guégan et al., 2018; Itoh et al., 2018; Strand, 2018; Deutscher et al., 2019; Huang et al., 2020; Wang et al., 2020). Due to their importance, any change in the composition or abundance of the bacterial species associated with the insect hosts may have an impact on their physiology. For example, several studies on fruit fly species, which are target for sterile insect technique applications, have shown that changes in the composition and abundance of gut-associated bacterial species may be triggered by irradiation treatments, which in turn may be related to physiological changes affecting the biological quality and the overall fitness of the host (Ami et al., 2010; Gavriel et al., 2010; Cai et al., 2018; Asimakis et al., 2019; Woruba et al., 2019).

However, the biological quality and the ecological fitness of irradiated insects can be restored through probiotic applications as has been shown in two major agricultural tephritid pest species, the Mediterranean fruit fly *Ceratitis capitata* and the Oriental fruit fly *Bactrocera dorsalis* (Niyazi et al., 2004; Ami et al., 2010; Yuval et al., 2013; Augustinos et al., 2015; Kyritsis et al., 2017; Cai et al., 2018). Ben-Ami et al. (2010) showed that, upon irradiation, the abundance of *Klebsiella oxytoca* decreased in the guts of Mediterranean fruit fly. However, the provision of *Klebsiella oxytoca* as probiotic supplement into the adult diet of irradiated medflies resulted in its successful colonization in the gut, thus enhancing the mating competitiveness and longevity of sterile males (Ami et al., 2010; Gavriel et al., 2010). Similarly, Cai et al. (2018) also found that radiation of *Bactrocera dorsalis* induced changes in the gut-associated microbiota resulting to a significant reduction in the ecological fitness. However, probiotic applications of the gut associated *Klebsiella oxytoca* (BD177 strain) were able to rescue these effects and restore the biological quality of irradiated insects.

During the last two decades, symbiotic bacteria have been harnessed for the control of mosquito vector species and mosquito-borne diseases (Flores and O’Neill, 2018; Guégan et al., 2018; Nazni et al., 2019; Zheng et al., 2019; Caputo et al., 2020; Crawford et al., 2020; Huang et al., 2020; Ryan et al., 2020). In parallel, several studies have focused on the characterization of mosquito gut-associated microbiota with an emphasis on bacterial species (for recent reviews see Guégan et al., 2018; Strand, 2018; Scolari et al., 2019). Environmental factors and food resources have been shown to play an important role in the acquisition of bacterial species from breeding sites, which in turn may determine the composition and abundance of bacterial species in the mosquito gastrointestinal tracts (Pastoris et al., 1989; Lindh et al., 2005; Rani et al., 2009; Wang et al., 2011, 2018; Minard et al., 2013a, b; Guégan et al., 2018; Chen et al., 2020; Saab et al., 2020). Mosquito host species, sex and age may also affect the structure of the gut bacteriome and the density levels of its associated bacterial species (Minard et al., 2014, 2018; Guégan et al., 2018; Mancini et al., 2018; Rosso et al., 2018; Wang et al., 2018; Chen et al., 2020). It is also worth noting that many studies have indicated that mosquito-associated bacterial species may affect both metabolism and life history traits including food digestion, supply of vitamins and amino acids, body size, oviposition site choice and egg production, longevity, sex ratio, larval development as well as virus dissemination (Chouaia et al., 2012; Mitraka et al., 2013; Sharma et al., 2013; Coon et al., 2014, 2016a, 2016b; Muturi et al., 2016; Dickson et al., 2017; Guégan et al., 2018).

*Aedes albopictus* (Diptera: Culicidae) is widespread all over the world contributing to the transmission of dengue, Zika and chikungunya viruses (Gasperi et al., 2012; Weaver et al., 2018). The Insect Pest Control Laboratory of the Joint FAO/IAEA Centre of Nuclear Techniques in Food and Agriculture has been developing the SIT package as a component of area-wide integrated pest management (AW-IPM) approaches to suppress *Ae. albopictus* populations. Previous studies suggested that a radiation dose (>35 Gy) may negatively affect the longevity and mating competitiveness of *Ae. albopictus* adult males (Bellini et al., 2013; Madakacherry et al., 2014; Zhang et al., 2016; Du et al., 2019). However, and given the importance of gut-associated bacterial species for the overall ecological fitness and biological quality of their hosts, none of these studies investigated whether the radiation affects the structure of the gut-associated bacteriome, and in the case of a negative effect, whether probiotic applications could restore the ecological fitness as previously observed for other pest species, for example, the tephritid *B. dorsalis* (Cai et al., 2018). In the present study, *Ae. albopictus* pupae were exposed at a radiation dose of 40 Gy, a dose which can induce full sterility in females and up to 99% sterility in males (Balestrino et al., 2010; Yamada et al., 2014; Zhang et al., 2015a, 2016; Du et al., 2019). The effect of radiation on the composition and density levels of the gut-associated bacterial species at the pupal stage as well as at 1- and 4-day-old males and females were studied using 16S *rRNA* gene-based next generation sequencing (NGS) and quantitative PCR (qPCR) approaches. The data produced were also assessed in respect to the age and sex, and they are discussed in the context of mating competitiveness of sterile males and potential microbiota manipulations for the enhancement of SIT applications against the major mosquito vector species *Ae. albopictus*.

## Materials and Methods

### Mosquito Strains and Maintenance

The experiments were conducted at the Joint FAO/IAEA Insect Pest Control Laboratory (hereafter IPCL), Seibersdorf, Austria. *Ae. albopictus* wild type strain (Guangzhou, China), known as GUA strain (Zhang et al., 2015b), at F_13_ generation was used in these experiments. Egg hatching, larvae rearing, and adult maintenance was performed as described previously (Zhang et al., 2015b). The colony was maintained under at 26 ± 1°C with a light: dark cycle of 12 h: 12 h, and 60 ± 10% relative humidity.

### Irradiation Procedures and Gut Dissection

Bovine-defibrinated blood meals were preheated and then provided to GUA females 7–9 days post emergence. Two days after the blood meal, a plastic 250-ml beaker containing 100 ml sterilized deionized water and a strip of sterilized white filter paper (white creped papers IF C140, Industrial Filtro S.r.l., Cologno Monzese, Italy) was placed in the cage (30 × 30 × 30 cm, BugDorm 1, MegaView, Taichung, Taiwan, China). After maturation, eggs were hatched as described previously (Zhang et al., 2015b). Larvae were fed on modified IAEA liquid larvae diet (Balestrino et al., 2014). Male and female pupae from 24 to 36 h after pupation were separated using an improved Fay and Morlan’s separator (Focks, 1980) and then were irradiated. The irradiation treatment was conducted with a Co-60 Gammacell irradiator 220 (Atomic Energy of Canada Ltd., Canada). The irradiation dose was set to 40 Gy, and the actual dose was measured with Gafchromic^®^ MD-V3 film (Ashland, Bridgewater, New Jersey, United States) and DoseReader 02 (FWT-92D, Far West Technology, Inc., Goleta, Canada). The irradiation dose was set to 40 Gy, a dose which has been shown to induce over 99% sterility on *Ae. albopictus* males and a reduction in longevity and mating competitiveness (Balestrino et al., 2010; Zhang et al., 2015a, 2016; Du et al., 2019). Due to time and space limitation, materials for pupae and adults were reared and irradiated separately. To minimize variation, pupae and adults of the same batch were taken as control group, respectively.

After irradiation, some of the pupae were immediately dissected under the stereomicroscope to isolate the whole gut to understand how irradiation may affect the gut-associated bacterial community. The rest of the pupae were reared separately in sterilized deionized water and placed in cage for adult emergence. Non-fed early emerged adults (both males and females) were collected and their whole gut was dissected upon adult emergence (less than 24 h) in order to see whether potential changes occurred in the pupal gut-associated bacteriome due to radiation could last until the adult stage. The rest of adults were supplied with 10% sucrose solution. To study whether any potential changes in the gut-associated bacteriome due to radiation are naturally restored during the first days of the adulthood, which is important for mating competitiveness and SIT applications, the whole guts from males and females were collected on the 4th day after emergence. Ventral diverticulum and Malpighian tubules were removed from every gut sample.

Samples from 12 groups: male pupae irradiated (MPI), male pupae control (MPC), female pupae irradiated (FPI), female pupae control (FPC), early emerged males irradiated (1DMI), early emerged males control (1DMC), early emerged females irradiated (1DFI), early emerged females control (1DFC), 4 days old males irradiated (4DMI), 4 days old males control (4DMC), 4 days old females irradiated (4DFI), 4 days old females control (4DFC), were collected for dissection. The detail information on the age, diet, sex and radiation treatment of the analyzed samples is shown in Supplementary Table 1. Alive adults which had been anesthetized at 4°C or alive pupae were surface disinfected by dipping in 70% ethanol for 1 min, placed into sterile 1 × PBS (phosphate buffer saline) for rinsing, and then dissected in sterile PBS under a binocular microscope with sterilized needles to get whole guts.

### DNA Extraction and 16S *rRNA* Gene Sequencing

Ten guts per tube were mechanically homogenized using sterile pestles in liquid nitrogen. DNA was extracted following the protocol of DNeasy Blood and Tissue Kit (QIAGEN, Germany), then the contents of two tubes were mixed thus each sample was consisting of 20 guts. DNA was concentrated to > 15 ng/μl according to the original concentration estimated by a NanoDrop 3,000 spectrophotometer and was used for next generation sequencing (NGS) and qPCR analysis. In summary, each group contained four replicates and each replicate included 20 guts.

The NGS analysis was based on the 16S *rRNA* gene. Two regions of the gene were amplified using the primers U341F (5′-CCTACGGGRSGCAGCAG-30) and 805R (50-GTGCCAGCM GCCGCGGTAA-3′) (V3-V4) and 909F (50-ACTCAAAK GAATWGACGG-30) and 1391R (5′-GACGGGCGGTGWG TRCA-3′) (V6-V8), respectively (Sogin et al., 2006; Cole et al., 2007; Eid et al., 2009; Klindworth et al., 2013). The PCRs, the preparation of the libraries and the sequencing using the Illumina MiSeq platform were performed by Macrogen (Macrogen, Seoul, Korea). The sequences have been deposited to NCBI under the accession number PRJNA682321.

### Bioinformatic Analysis

De-multiplexing of the raw sequencing reads was performed followed by their conversion to FASTQ, and standard algorithms were used to trim the Illumina adapters. The sequence reads were prepared for subsequent analysis in usearch v.10 and v.11 (Edgar, 2017). The paired-end reads were assembled and trimmed by length, and the usearch -fastq_mergepairs option in usearch v.11 was used to check for errors and assess quality. Unassembled reads as well as reads which were outside the range of 400–520 bp were not included in the downstream analysis. Using the -fastq_filter in usearch v.11, the quality of the assembled sequences was further improved while unique read sequences and abundances were found with the -fastx_uniques option. The -cluster_otus command was used for the clustering of sequences into operational taxonomic units (OTUs) at 97% similarity (Edgar, 2013), while the -unoise3 option of usearch v.10 was used to remove chimeras (Edgar, 2016b). Taxonomy was assigned against the SILVA 128 release database (Quast et al., 2012; Edgar, 2016a) using the qiime feature-classifier classify-consensus-blast command of qiime2-2020.11 with 0.97% identity (Bolyen et al., 2019; Estaki et al., 2020). The OTUs of chloroplast, Archaea and unassigned OTUs were manually removed and the taxonomy was repeated as mentioned above. No mitochondrial OTUs were detected.

Relative abundance heatmap was created with matrics display in PRIMER version 7 + and was based on the OTU table along with the taxonomy data previously obtained in order to visualize the most dominant OTUs (*n* = 35) in each sample group at phylum and genus levels. To test the overall statistical differences between the different factors, the bootstrap averages were calculated using the Bray Curtis resemblance matrix of the OTUs abundance results which is based on the analysis of similarity (ANOSIM) pairwise test and plotted in PRIMER version 7 + using number of bootstraps between 50 and 150 per sample.

### Quantitative Analysis of the Main Gut Bacteria Groups

Based on the NGS results, the three most abundant genera, which also varied greatly among treatments, were quantified by quantitative PCR (qPCR): *Aeromonas*, *Elizabethkingia* and *Enterococcus*. The V3-V4 region of the 16S *rRNA* gene sequences of these three genera obtained from the NGS analysis were used for BLAST searches in the databases to search the most similar reference sequences. All available 16S *rRNA* gene sequences of the gut-associated bacteria of the *Ae. albopictus* GUA strain detected in our previous study (Chen et al., 2020) were also taken as reference for primer specificity. Pairs of genus-specific qPCR primers were designed for these three genera with Primer3plus^1^. Primer self-complementarity were checked with NCBI primer-blast. We also searched the Silva database to ensure that our primer pairs fit as many target sequences whereas as few untargeted ones. The *Ae. albopictus* ribosomal protein S6 (*rpS6*) gene was chosen as reference. The primers used in this analysis along with the annealing temperatures (TA) and melting temperature (TM) are presented in Supplementary Table 2. The amplification was performed using iQ^TM^ SYBR^®^ Green Supermix (Bio-Rad, United States). The reaction mixture (15 μl) consisted of 5 ng DNA template and 7.5 μl of 2 × Supermix. qPCR and 200 nM of each primer was performed with a CFX96 Touch Real-Time PCR Detection system (Bio-Rad, United States). The concentration of DNA was diluted, and 5 ng DNA was used in 15 μl reactions. Due to different T(A)s of the primers, housekeeping gene *rpS6* and target genes were put on the same wells of different plates. An initial denaturation at 95°C for 2 min was followed by 40 cycles consisting of denaturation at 95°C for 10 s, at annealing temperature for 60 s. The fluorescence collection was performed at the annealing stage. To check and confirm the quality of amplification, a melting profile was generated for the amplicon over a temperature range of 65–95°C. Melting curves for each sample were analyzed after each run to check that there was no primer dimer formation. Four biological replicates were performed for each sample. There were three technical replicates for each biological sample. Relative quantity (Mean ± SEM) of products was calculated using CFX Manager^TM^ Software (Bio-Rad Laboratories, Inc.). The relative abundance of bacteria was determined by using the 2^−ΔΔ*CT*^ calculation method.

### Statistical Analysis

Normalcy of the data was assessed by the D’Agostino-Pearson omnibus normality test of the GraphPad Prism 6.0 software. For the NGS results, alpha-diversity analysis was also performed with Qiime2 and usearch v.11. Pielou.s.evenness and Richness index were used to estimate the number of OTUs per samples whereas Shannon’s and Simpson’s reciprocal [1/Simpson’s Index (D)] indices were used to determine species diversity. The bacterial richness and diversity indices among the samples were compared using Kruskal-Wallis test and Dunn’s multiple comparisons test with the default setting or Two-tailed Mann-Whitney *U*-test based on different classified groups. Similarities in the structure of bacterial communities and the role of different factors such as age, sex and radiation were assessed using the metric multidimensional scaling (mMDS) plot with bootstrap averages in PRIMER version 7 + and were displayed with a Bray and Curtis matrix based on the square-root transformation of the bacterial OTU abundance data (Clarke and Gorley, 2016). The tests were based on the multivariate null hypothesis via the use of the non-parametric statistical method PERMANOVA (Anderson, 2001).

For qPCR results, Dunn’s multiple comparisons test or Two-tailed Mann-Whitney *U*-test was used to compare the relative density of the selected bacterial species.

## Results

The sequence data of the two 16S *rRNA* gene regions, amplified by the primer pairs U341F/805R and 909F/1391R, were compared and the analysis indicated that there were no statistically significant differences (Supplementary Table 3). Based on this finding, the sequence data of these two regions were combined and used for all the downstream analysis which resulted to the data, figures and tables presented below.

### Effect of Age, Sex, and Radiation on the Alpha Diversity of Gut Microbiota

The effects of age, sex and radiation on the bacterial community composition and diversity in the guts of *Ae. albopictus* GUA strain, including male and female pupae, 1- and 4- day-old male and female adult mosquitoes, were investigated by 16S *rRNA* gene sequencing. A total of 506,854 (minimum 30433–maximum 46819) reads, corresponding to 496 (minimum 30–maximum 53) OTUs, were used for analysis in this study after quality filtering of the sequencing results (Table 1 and Supplementary File 1).

**TABLE 1:** Analysis of α diversity indices of the experimental samples.

**ID**	**No. of reads (min-max)**	**No. of OUTs (min-max)**	**Pielou.s.evenness**	**Species richness**	**Species diversity indices**	
					**Shannon**	**Simpson reciprocal**
MPI	39574 (33521–46250)	53 (50–55)	0.68 ± 0.04 a	52.75 ± 1.11 a	2.70 ± 0.15 a	0.87 ± 0.02 a
MPC	38066 (34262–42291)	49 (45–56)	0.71 ± 0.06 a	49.25 ± 2.39 ac	2.77 ± 0.23 a	0.89 ± 0.04 a
FPI	30433 (25015–38219)	45 (40–49)	0.72 ± 0.07 a	45.25 ± 2.06 ad	2.75 ± 0.29 a	0.87 ± 0.03 a
FPC	36737 (31621–41777)	44 (40–48)	0.69 ± 0.09 a	44.25 ± 1.65 ae	2.64 ± 0.35 a	0.85 ± 0.06 a
1DMI	45956 (44453–47533)	42 (37–45)	0.58 ± 0.02 ac	41.72 ± 2.00 bdef	2.16 ± 0.08 ac	0.84 ± 0.01 ac
1DMC	43916 (38650–47109)	50 (46–53)	0.63 ± 0.04 a	50.00 ± 1.47 af	2.46 ± 0.18 a	0.85 ± 0.02 ac
1DFI	41387 (30377–48489)	43 (29–50)	0.65 ± 0.06 a	42.99 ± 4.75 af	2.44 ± 0.26 a	0.86 ± 0.02 a
1DFC	45642 (40658–48208)	34 (25–40)	0.51 ± 0.01 ab	33.50 ± 3.18 bf	1.80 ± 0.07 ab	0.75 ± 0.02 ab
4DMI	46819 (45618–48200)	30 (25–34)	0.39 ± 0.02 b	29.71 ± 1.89 bf	1.30 ± 0.05 b	0.61 ± 0.01 b
4DMC	45925 (42468–47963)	31 (26–38)	0.41 ± 0.01 b	30.69 ± 2.62 bef	1.40 ± 0.04 bc	0.66 ± 0.01 bc
4DFI	46604 (46000–47354)	37 (35–41)	0.40 ± 0.02 bc	37.00 ± 1.35 bdef	1.46 ± 0.08 bc	0.64 ± 0.03 b
4DFC	45797 (45098–46758)	39 (38–40)	0.37 ± 0.02 b	39.00 ± 0.41 bcd	1.37 ± 0.06 bc	0.60 ± 0.01 b

*For each diversity index, Kruskal-Wallis test followed by the Dunn’s multiple comparisons test, (P < 0.05). Significant differences are indicated by different letters based on the data presented in Supplementary Tables 4, 5. Data are presented as Mean or Mean ± SEM of four biological replicates per sample.*

**FIGURE 1:**
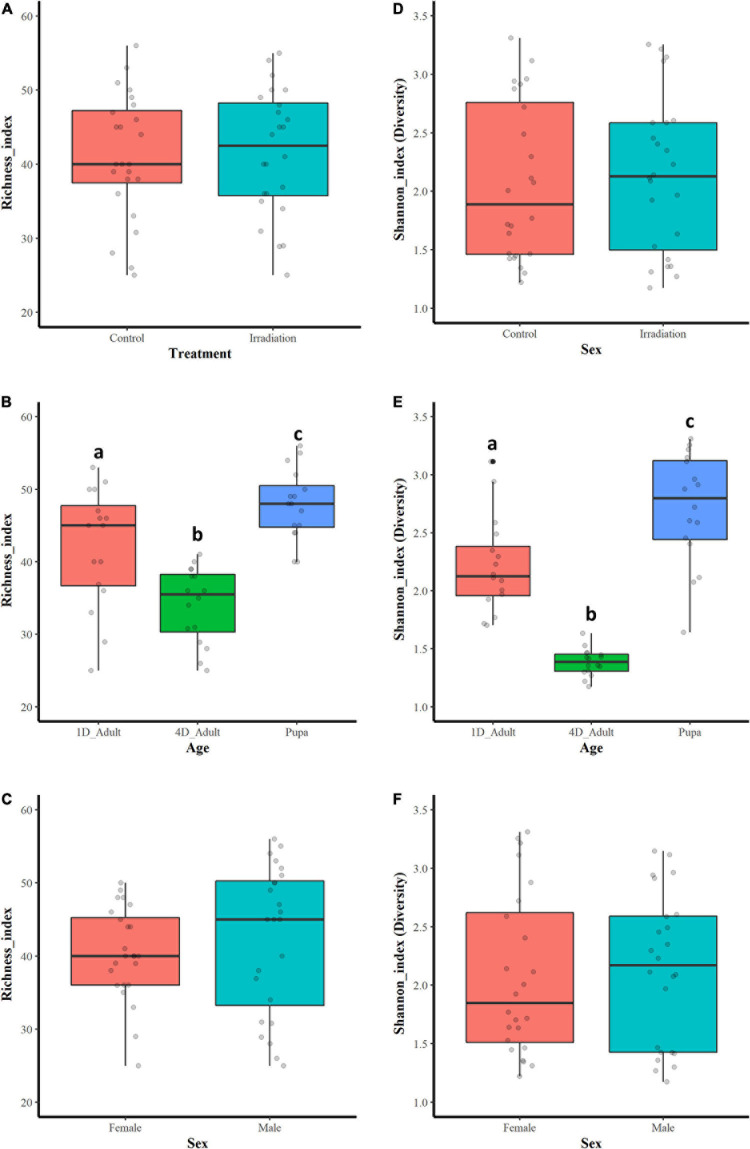
Bacterial community richness and diversity based on the Species richness and Shannon indices. Bacterial richness **(A)** and diversity **(D)** of guts between irradiated and non-irradiated samples. Bacterial richness **(B)** and diversity **(E)** of guts from pupa, 1- and 4-day-old adults. Bacterial richness **(C)** and diversity **(F)** of guts between male and female mosquitoes. Boxes extend between the 25th and 75th percentile. A thick line denotes the median. The whiskers extend up to the most extreme values. The gray circles indicate the number of data points used in each plot. Two-tailed Mann-Whitney *U*-test was used to compare the bacterial richness and diversity indices in treatment or sex groups. Kruskal-Wallis test and Dunn’s multiple comparisons test were used to compare the bacterial richness and diversity indices in age groups. Significant differences are indicated by different letters.

Among all the sequenced samples, significant difference on the bacterial community abundance was observed based on species richness indices (Kruskal-Wallis test, *df* = 11, *X*^2^ = 37.15, *P* < 0.01) (Table 1). The samples were then classified based on treatment (irradiated or non-irradiated), age (pupa, 1- and 4-day-old) and sex (male and female), and, respectively compared based on richness and Shannon indices. The results showed that there were no significant differences on bacterial richness in treatment (Figure 1A, Two-tailed Mann-Whitney *U*-test, *U* = 296.5, *P* = 0.869) or sex (Figure 1C, Two-tailed Mann-Whitney *U*-test, *U* = 335, *P* = 0. 0.337), however, significant difference was observed on age with the highest bacterial diversity being observed in the guts of pupae and those of 4-day-old adults (Figure 1B, Kruskal-Wallis test and Dunn’s multiple comparisons test, *x*^2^ = 23.451, *df* = 2, *P* < 0.001). Using the Pielou.s.evenness index for assessing the diversity along with species richness, significant differences were detected between samples (*x*^2^ = 37.347, *df* = 11, *P* < 0.001).

Regarding the bacterial community diversity, significant differences were observed in the tested samples according to the Shannon (Kruskal-Wallis test, *x*^2^ = 37.390, *df* = 11, *P* < 0.001) and Simpson indices (Kruskal-Wallis test, *x*^2^ = 35.737, *df* = 11, *P* < 0.0001) (Table 1). Samples were classified as above-mentioned and comparison was performed based on Shannon index. As shown in Figure 1E, the bacterial diversity was highest in the guts of pupae, followed by 1- and 4-day-old adults (Kruskal-Wallis test and Dunn’s multiple comparisons test, *x*^2^ = 34.839, *df* = 2, *P* < 0.0001). No significant differences on bacterial diversity of guts with respect to irradiation treatment (Figure 1D, Two-tailed Mann-Whitney *U*-test, *U* = 301.5, *P* = 0.798) and sex (Figure 1F, Two-tailed Mann-Whitney *U*-test, *U* = 298, *P* = 0.846) were observed (Supplementary File 1).

### Major Bacterial Taxa

**FIGURE 2:**
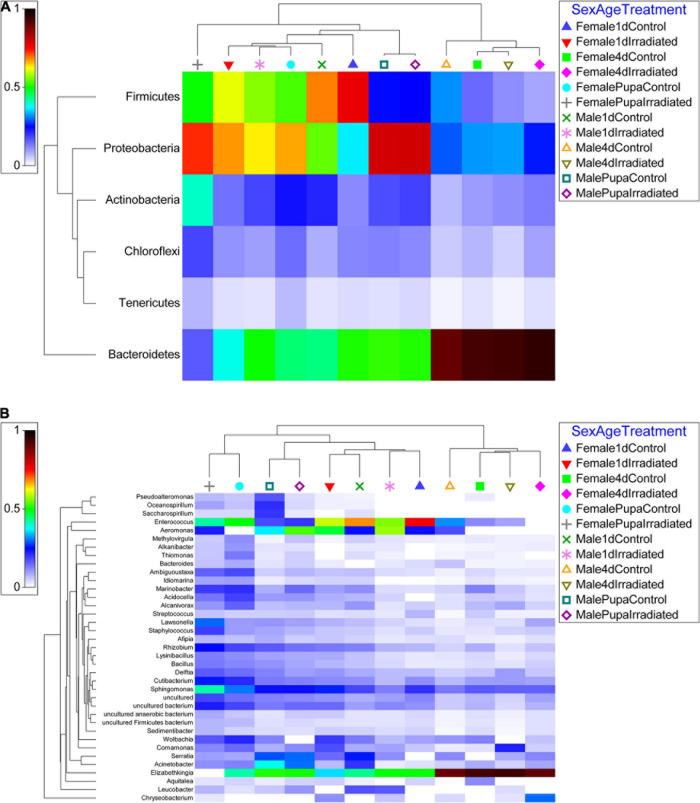
Relative abundance heatmap of the most dominant phylum and genus of all samples examined. **(A)** Phylum level. **(B)** Genus level. OTUs abundance results from qiime2 analysis were transformed with square root transformation and averaged based on the number of replicates per sample. The resemblance matrix was conducted with Bray Curtis similarity of the OTUs abundance results.

Based on the NGS results, the most abundant gut bacteria (≥ 2%) in *Ae. albopictus* GUA strain are shown in Figure 2. Based on phylum, the abundance of OUTs was relatively uniform, but also showed some minor differences, in all tested samples (Figure 2A). *Bacteroidetes* (18.9 and 21.1%), *Firmicutes* (16.4 and 43.4%), *Proteobacteria* (55.6 and 31.7%) and *Actinobacteria* (6.9 and 2.7%), were the four most abundant phyla in pupae and 1-day-old adult mosquitoes, respectively, however, *Bacteroidetes*, with a relative abundance of up to 86.0%, was the main phylum in the guts of 4-day-old adults. These differences were also observed at the genus level. For example, *Elizabethkingia* was the genus with the highest relative abundance in 4-day-old adults while *Elizabethkingia* and *Enterococcus* were the two relatively high abundant genera in pupae and 1-day-old mosquitoes. In addition, except for female pupae, *Aeromonas* was also occurred with a high relative abundance in male pupae and 1-day-old mosquitoes. Interestingly, when compared to the corresponding non-irradiated (control) mosquitoes, the irradiated samples exhibited a higher average abundance of *Aeromonas* (Figure 2B). In contrast, except for 1-day-old male mosquitoes, the average abundance of *Enterococcus* in the irradiated mosquitoes was lower than their corresponding control samples (Figure 2B).

### Effect of Age, Sex, and Radiation on the Beta Diversity of Gut Microbiota

**FIGURE 3:**
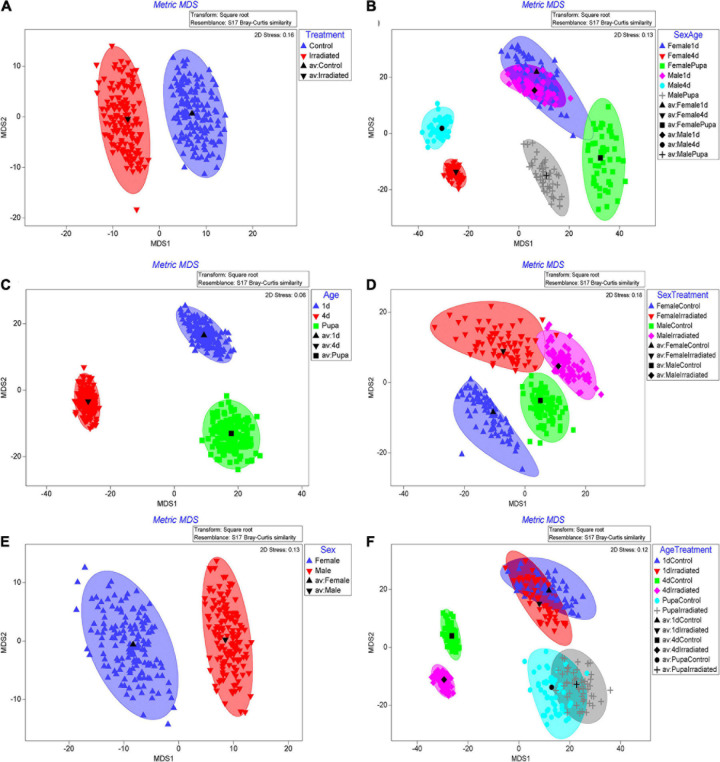
Bootstrap averages with Metric multidimensional scaling (mMDS) of bacterial communities based on relative abundances of OTUs originating from treatment, age or sex groups **(A–C)** or combinations of them **(D–F)**. OTUs abundance results from qiime2 analysis were transformed with square root transformation and the resemblance matrix was conducted with Bray Curtis similarity. Bootstrap averages were analyzed with 5 **(D,F)**, 75 **(E)** and 150 **(A–C)** bootstraps per sample indicated by the number of points in the plots.

Regarding the beta diversity of the bacterial communities, the sequenced samples were classified according to irradiation treatment (hereafter treatment), age or sex. The results clearly revealed that bacterial communities differed significantly in respect to these three independent factors (Table 2, PERMANOVA; Treatment: *P* = 0.004; Age: *P* = 0.001; Sex: *P* = 0.001). The metric multidimensional scaling (mMDS) showed that unique community clusters were formed separately between the irradiated and non-irradiated samples (Figure 3A). Similarly, the formation of distinct clusters among pupae, 1- and 4-day-old adults (Figure 3B) or between male and female mosquitoes (Figure 3C) were, respectively observed.

**TABLE 2:** PERMANOVA table of results for all three factors and their combinations for genera level abundance.

	***df***	**SS**	**MS**	**Pseudo-F**	**P (perm)**	**Unique perms**
Treatment	1	1898.8	1898.8	3.8904	**0.004**	998
Age	2	24,882	12,441	25.49	**0.001**	999
Sex	1	3,086	3,086	6.3228	**0.001**	998
Treatment × Age	2	1277.2	638.62	1.3084	0.219	998
Treatment × Sex	1	1063.8	1063.8	2.1795	0.06	998
Age × Sex	2	3156.7	1578.4	3.2338	**0.001**	999
Treatment × Age × Sex	2	1416.1	708.04	1.4507	0.156	999
Res	36	17,571	488.08			
Total	47	54,351				

*Twelve samples were studied with four biological replicates each. Within the table, statistically significant differences (P < 0.05) can be seen in bold values in all three factors separately and in the combination of age and sex. Perm(s) = permutations.*

Strong clustering of bacterial communities was observed only when age and sex factors combined (Table 2, PERMANOVA; Age × Sex: *P* = 0.001). Results of mMDS confirmed the clusters (Figures 3D–F). However, three factor combinations did not show such a high clustering (Table 2, PERMANOVA, *P* = 0.156). In addition, the combination between Age and Sex factors or Treatment and Sex factors also did not present high clustering (Table 2, PERMANOVA, Treatment × Age: *P* = 0.219, Treatment × Sex: *P* = 0.060).

### Relative Abundance of *Aeromonas, Elizabethkingia*, and *Enterococcus*

**FIGURE 4:**
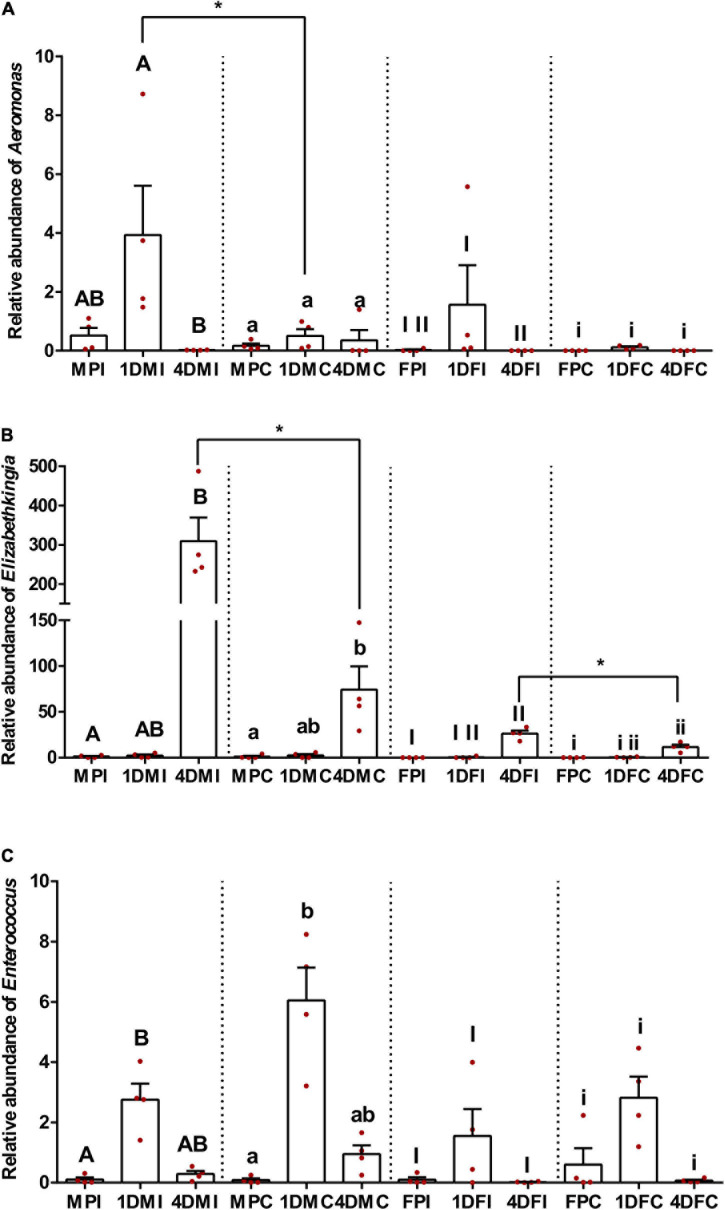
Relative abundance of three major bacterial groups based on qPCR. **(A)** *Aeromonas*; **(B)** *Elizabethkingia*; **(C)** *Enterococcus*. Dots represent biological replicates, each one as the mean of three technical replicates. Relative abundance data (*n* = 4 for each sample, Mean ± S.E.M) are presented relative to the housekeeping gene *rps6*. Within **(A–C)** samples of the same sex and irradiation/control, values followed by different lowercase letters or capital letters or Roman numbers were statistically different using Kruskal-Wallis test and Dunn’s multiple comparisons test (*P* < 0.05). Two-tailed Mann-Whitney *U*-test was performed between the irradiated and control samples of the same developmental stage/age/sex. Only the significantly different results are shown in the figures. * indicates *P* < 0.05.

Based on qPCR results, the relative abundance of the three taxa is presented in Figure 4. In general, all three bacterial taxa showed clear and distinct patterns among different developmental stages, ages, and sexes. The relative abundance of *Aeromonas* in the midgut of pupae, with or without irradiation, showed no significant difference compared to either 1- or 4-day-old adult mosquito for both males and females (Dunn’s multiple comparisons test, *P* > 0.05) except for the female pupae which exhibited a lower density than 1-day-old female adults (Dunn’s multiple comparisons test, *P* < 0.05). Interestingly, irradiated 1-day-old adult mosquitoes had a higher Aeromonas density than in 4-day-old mosquitoes (Dunn’s multiple comparisons test, *P* < 0.05), but this pattern was not observed in the control groups (Dunn’s multiple comparisons test, *P* > 0.05). One-day-old adult mosquitoes showed no significant difference regarding *Elizabethkingia* abundance compared to either pupae or 4-day-old males and females, irrespective to irradiation treatment (Dunn’s multiple comparisons test, *P* > 0.05). However, 4-day-old adults had a higher *Elizabethkingia* density than their respective pupae (Dunn’s multiple comparisons test, *P* < 0.05). No significant difference on *Enterococcus* density was observed among pupae, 1- and 4-day-old female adults, regardless of irradiation treatment (Dunn’s multiple comparisons test, *P* > 0.05). In respect to male mosquitoes, 1-day-old male adults exhibited higher *Enterococcus* density than pupae (Dunn’s multiple comparisons test, *P* < 0.05). Four-day-old males showed no significant difference compared to either pupae or 1-day-old males (Dunn’s multiple comparisons test, *P* > 0.05). When combined the data from same sex, males had a higher *Aeromonas* density than females in both treatment and control groups (Two-tailed Mann-Whitney *U*-test, *P* < 0.05), but this was not observed for *Elizabethkingia* (Two-tailed Mann-Whitney *U*-test, *P* > 0.05) or *Enterococcus* (Two-tailed Mann-Whitney *U*-test, *P* > 0.05).

The effect of irradiation on the abundance of the three bacterial taxa was compared to the respective control groups of the same sex and developmental stage/age. Increased *Aeromonas* density was only observed in the irradiated 1-day-old adult males when compared to non-irradiated ones (Two-tailed Mann-Whitney *U*-test, *P* < 0.05). Similar results were found in 4-day-old irradiated adults which had higher *Elizabethkingia* density than the controls in both males and females (Two-tailed Mann-Whitney *U*-test, *P* < 0.05). No significant difference was observed on the *Enterococcus* density between irradiated and non-irradiated samples (Two-tailed Mann-Whitney *U*-test, *P* > 0.05). Detailed information about the statistical analysis is shown in Supplementary Tables 4, 5.

## Discussion

Sterile insect technique (SIT), as a component of area-wide integrated pest management (AW-IPM) programmes, has been successfully applied to suppress or even locally eradicate insect populations of several major agricultural and livestock pest species during the last seventy years (Vreysen et al., 2007; Dyck et al., 2021). Based on these successful applications, recent efforts have focused on the development and validation of the SIT package to suppress populations of mosquito disease vectors including *Ae. albopictus* and *Ae. aegypti* (Lees et al., 2015; Bourtzis et al., 2016; Bouyer and Vreysen, 2020). Indeed, several small-scale field trials have provided quite encouraging results suggesting that SIT has the potential to suppress *Aedes* mosquito populations (Bellini et al., 2013; Kittayapong et al., 2018, 2019; Zheng et al., 2019). One of the key elements for the successful application of SIT to suppress populations of insect pests and disease vectors, including mosquitoes, is the production of high-quality sterile males (Bouyer and Vreysen, 2020; Dyck et al., 2021).

It has been shown that mass rearing, handling, transport, release and sterilization may affect the male mating competitiveness (Bellini et al., 2013; Madakacherry et al., 2014; Maïga et al., 2014; Yamada et al., 2014; Diallo et al., 2019; Du et al., 2019; Zheng et al., 2019; Bouyer and Vreysen, 2020; Dyck et al., 2021). In addition, several studies have suggested that changes in the male mating competitiveness may be associated with radiation-induced changes in the gut-associated bacteriome of SIT target species (Ami et al., 2010; Gavriel et al., 2010; Cai et al., 2018; Asimakis et al., 2019; Woruba et al., 2019). Using 16S *rRNA* gene-based sequencing approaches, the present study investigated the potential effect of age, sex and radiation on the gut-associated bacteriome of the laboratory-reared *Ae. albopictus* GUA strain. Our results clearly showed that all three factors can affect the gut-associated bacterial communities with age having the highest impact triggering significant changes on bacterial diversity and clustering among pupae, 1- and 4-day-old adult samples. It is also worth noting that the qPCR results revealed that the relative density levels of *Aeromonas* are higher in male samples compared to all other samples and that the irradiation triggers an increase in the density levels of both *Aeromonas* and *Elizabethkingia* in the mosquito gut at specific stages.

Previous studies on fruit flies have clearly shown that age plays an important role in shaping their gut-associated bacteriome (Ami et al., 2010; Hamden et al., 2013; Augustinos et al., 2015; Malacrinò et al., 2018). The present study confirmed that age is a critical factor in shaping the bacterial communities in *Ae. albopictus* guts too (Figures 3C,D). *Actinobacteria*, *Bacteroidetes*, *Firmicutes* and *Proteobacteria* were found to be the four main phyla in pupae and 1-day-old adult mosquitoes while *Bacteroidetes* was detected as the main phylum in 4-day-old adults (Figure 2A). However, age-dependent differences may be reflecting the distinct feeding behaviors during different developmental stages. In Diptera insects, once larva becomes pupa, it does not feed anymore until adult emergence (Truman, 2019). This indicates that all the metabolic activities taking place during the pupal stage are supported from nutrients produced through gut bacterial-mediated decomposition of larva-acquired intestinal contents. It is interesting to note that during the dissection of mosquito pupal guts, partial remnants of intestinal contents were observed in some samples and this may explain why higher bacterial diversity was observed at the pupal stage compared to adults (Figure 1E). Teneral mosquito adults (<24 h old) do not feed and their guts are usually empty indicating that the intestinal contents have been digested after eclosion. Four-day-old adult mosquitoes are only provided with sugar solution which may explain why teneral adults may present lower bacteria diversity compared to pupae (Figure 1E). It is noteworthy that *Elizabethkingia* is the most abundant bacterium in 4-day-old adults (Figure 2B), which is in line with previous studies showing that this bacterial species is mainly involved in the digestion of blood and sugar (Wang et al., 2011; Chen et al., 2015, 2020).

Sex may also affect the composition of mosquito gut-associated bacteriomes and differences have mainly been attributed to different food sources and nutrients (Minard et al., 2013a, 2014; Mancini et al., 2018). For blood-sucking insects such as mosquitoes, females require animal blood, which contains essential nutrients for the maturation of eggs (Dodd, 1995; Barrozo, 2019). Intestinal bacterial species are involved in the digestion of blood to produce these essential nutrients and this may explain why the abundance of key bacterial species increases after a blood meal (Strand, 2018). Unlike females, males usually feed only on honey or sugar throughout their entire lifetime. Previous studies have documented clear differences between the bacterial communities associated with male and female mosquitoes (Minard et al., 2018; Rosso et al., 2018; Wang et al., 2018; Chen et al., 2020). The present study also confirmed the different structure of the gut-associated bacterial communities present in *Ae. albopictus* male and female mosquitoes (Figure 3C).

As in many insect species, *Ae. albopictus* is characterized by the phenomenon of protandry, male mosquitoes develop faster than female ones (Zhang et al., 2015b). In addition, it has been shown that *Aeromonas*, as well as *Klebsiella* and yeast, can accelerate the larval development in the mosquito species *Culex pipiens* (Díaz-Nieto et al., 2016). Interestingly, our study showed that *Aeromonas* bacteria are present in higher densities in *Ae. albopictus* males compared to females in both irradiation and control groups, while no difference was observed in respect to *Elizabethkingia* and *Enterococcus* taxa (Supplementary Table 2). These results suggest that *Aeromonas* maybe a contributing factor for the different developmental rate observed in *Ae. albopictus* males and females (Supplementary Table 2). Further experimental work is needed to test this hypothesis and see whether *Aeromonas* could be used as probiotic to further enhance the protandry phenomena in *Ae. albopictus* thus enhancing sex separation efficiency and male recovery in support of SIT applications against this major vector species.

Several studies have shown that radiation may affect the diversity and abundance of gut-associated bacteria in insects (Ami et al., 2010; Lauzon and Potter, 2012; Yuval et al., 2013; Cai et al., 2018; Asimakis et al., 2019; Woruba et al., 2019). However, our results suggest that irradiation of *Ae. albopictus* pupae does not affect the overall abundance and diversity of the gut-associated bacterial community (Table 2 and Figures 1A,D), but it rather triggers a shift to its actual composition (Table 2 and Figure 3). It is worth noting that 4-day-old non-irradiated mosquitoes have significant higher abundance of *Elizabethkingia* when compared to their irradiated counterparts (Supplementary Table 2), whereas this difference is not observed in pupae and 1-day-old mosquitoes. The *Elizabethkingia*’s genome is properly equipped to metabolize sugars present in the host intestine (Kukutla et al., 2014). This has been confirmed by an increase of *Elizabethkingia*’s density levels in the intestine of 14-day-old male *Ae. albopictus* adults, which have been fed on sugar solution only, by more than 30 times when compared to 1-day-old males (Chen et al., 2020). In addition, *Elizabethkingia* encodes an antioxidant protein HemS (heme-degrading protein), which is known to reduce the oxidative damage during blood digestion (Kukutla et al., 2014). Previous studies have shown that irradiation increases the abundance of oxygen free radicals in the intestine of insects causing oxidative damages (Zaghloul et al., 2013; Ali et al., 2017; Wang et al., 2019). Based on this observation, the higher abundance of *Elizabethkingia* detected in the irradiated adults in the present study may indicate that irradiated mosquitoes use this bacterium to remove the oxygen free radicals to reduce any potential oxidative damage. In addition, the genome of *Elizabethkingia* encodes for several hemolysins, and with their hemolytic activity this bacterial species can participate in the digestion of red blood cells (Kukutla et al., 2014). The sharp increase of the density levels of this bacterium in the guts of female mosquitoes after a blood meal is also in agreement with this (Chen et al., 2020).

In conclusion, the effects of age (pupa, 1- and 4-day-old adults), sex (female and male) and radiation on the gut-associated bacterial species of lab-reared *Ae. albopictus* were investigated in the present study using 16S *rRNA* gene-based next generation sequencing approaches. The results showed that age, perhaps in conjunction with the diet, is a key factor that can shape the diversity and structure of the mosquito gut-associated bacteriome. In addition, sex and radiation may also affect the bacteria community, even though no significant impact was observed on richness and diversity. In addition, our data suggested that *Aeromonas* could potentially be used as probiotics to enhance protandry and sex separation in support of SIT applications against *Aedes albopictus*, while the functional role of *Elizabethkingia* in respect to oxidative stress and damage in irradiated mosquitoes needs further investigation.

## Data Availability Statement

The datasets presented in this study can be found in online repositories. The names of the repository/repositories and accession number(s) can be found below: https://www.ncbi.nlm.nih.gov/, PRJNA682321 and https://dataverse.harvard.edu/dataset.xhtml?persistentId=, doi: 10.7910/DVN/HEL3PS.

## Author Contributions

DZ and SC performed the experiments, analyzed the data, and drafted the manuscript. AA-A performed the bioinformatic analysis, interpreted the data, contributed to the drafting, and critical revision of the manuscript. KB conceived the study, designed the experiments, interpreted the data, contributed to the drafting, and critically revised the manuscript. All authors approved the final version of the manuscript.

## Conflict of Interest

The authors declare that the research was conducted in the absence of any commercial or financial relationships that could be construed as a potential conflict of interest.

## Footnotes

1 http://www.bioinformatics.nl/cgi-bin/primer3plus/primer3plus.cgi
