# Supplementary material for: The Effect of Radiation on the Gut Bacteriome of Aedes albopictus
Source: Front Microbiol. 2021 Jul 8;12:671699. doi: 10.3389/fmicb.2021.671699 (PMC8299835; doi:10.3389/fmicb.2021.671699)
Supplement: Supplementary file 1 [file Presentation_1.pdf]

The effect of radiation on the gut bacteriome of Aedes albopictus

Dongjing Zhang, Shi Chen, Adly Abd-Alla and Kostas Bourtzis

25/04/2021

load data and needed packages

```
setwd("C:/Users/abdallaa/OneDrive - IAEA/My/Kostas_paper 2020/Final_version_F
eb_2021/Revission/raw_data")
alpha <- read.csv("Fig_1.csv")

str(alpha)

## 'data.frame':    48 obs. of  15 variables:
##  $ BarcodeName      : chr  "1_Aa_fe_1d_con_1_1" "2_Aa_fe_1d_con_2_1" "3_Aa
_fe_1d_con_3_1" "4_Aa_fe_1d_con_4_1" ...
##  $ Pielou.s.evenness: num  0.534 0.487 0.494 0.544 0.572 ...
##  $ Richness         : num  25 33 36 40 29 ...
##  $ Shannon          : num  1.72 1.7 1.77 2.01 1.93 ...
##  $ Simpson          : num  0.756 0.728 0.714 0.812 0.827 ...
##  $ Sex1             : chr  "2-Female" "2-Female" "2-Female" "2-Female" ...
##  $ Age1             : chr  "2-1d Adult" "2-1d Adult" "2-1d Adult" "2-1d Ad
ult" ...
##  $ Stage            : chr  "Adult" "Adult" "Adult" "Adult" ...
##  $ Treatment1       : chr  "2-Control" "2-Control" "2-Control" "2-Control"
...
##  $ SampleID         : int  1 1 1 1 2 2 2 2 3 3 ...
##  $ Age_Irradiation  : chr  "1_F_Control" "1_F_Control" "1_F_Control" "1_F_
Control" ...
##  $ Sample           : chr  "F1DC" "F1DC" "F1DC" "F1DC" ...
##  $ Treatment        : int  2 2 2 2 1 1 1 1 2 2 ...
##  $ Age              : int  2 2 2 2 2 2 2 2 3 3 ...
##  $ Sex              : int  2 2 2 2 2 2 2 2 2 2 ...

attach(alpha)
head(alpha)

##          BarcodeName Pielou.s.evenness Richness  Shannon   Simpson     Sex
1
## 1 1_Aa_fe_1d_con_1_1         0.5335210 25.00000 1.717338 0.7558498 2-Femal
e
## 2 2_Aa_fe_1d_con_2_1         0.4868625 32.99895 1.702319 0.7283268 2-Femal
e
## 3 3_Aa_fe_1d_con_3_1         0.4938661 35.99952 1.769779 0.7135968 2-Femal
e
## 4 4_Aa_fe_1d_con_4_1         0.5437077 39.99999 2.005672 0.8119299 2-Femal
```

```
e
## 5 5_Aa_fe_1d_ira_1_1         0.5717539 28.97187 1.925265 0.8270265 2-Femal
e
## 6 6_Aa_fe_1d_ira_2_1         0.6613343 50.00000 2.587155 0.8626072 2-Femal
e
##         Age1 Stage   Treatment1 SampleID Age_Irradiation Sample Treatment
Age
## 1 2-1d Adult Adult    2-Control        1     1_F_Control   F1DC         2
2
## 2 2-1d Adult Adult    2-Control        1     1_F_Control   F1DC         2
2
## 3 2-1d Adult Adult    2-Control        1     1_F_Control   F1DC         2
2
## 4 2-1d Adult Adult    2-Control        1     1_F_Control   F1DC         2
2
## 5 2-1d Adult Adult 1-Irradiated        2  1_F_Irradiated   F1DI         1
2
## 6 2-1d Adult Adult 1-Irradiated        2  1_F_Irradiated   F1DI         1
2
##   Sex
## 1   2
## 2   2
## 3   2
## 4   2
## 5   2
## 6   2
```

```
alph=na.omit(alpha)
alph
```

```
##             BarcodeName Pielou.s.evenness Richness  Shannon   Simpson     S
ex1
## 1   1_Aa_fe_1d_con_1_1         0.5335210 25.00000 1.717338 0.7558498 2-Fem
ale
## 2   2_Aa_fe_1d_con_2_1         0.4868625 32.99895 1.702319 0.7283268 2-Fem
ale
## 3   3_Aa_fe_1d_con_3_1         0.4938661 35.99952 1.769779 0.7135968 2-Fem
ale
## 4   4_Aa_fe_1d_con_4_1         0.5437077 39.99999 2.005672 0.8119299 2-Fem
ale
## 5   5_Aa_fe_1d_ira_1_1         0.5717539 28.97187 1.925265 0.8270265 2-Fem
ale
## 6   6_Aa_fe_1d_ira_2_1         0.6613343 50.00000 2.587155 0.8626072 2-Fem
ale
## 7   7_Aa_fe_1d_ira_3_1         0.8084004 47.00000 3.112461 0.9206851 2-Fem
ale
## 8   8_Aa_fe_1d_ira_4_1         0.5594015 45.99999 2.141748 0.8178528 2-Fem
ale
## 9   9_Aa_fe_4d_con_1_1         0.3998358 39.00000 1.464823 0.6191617 2-Fem
ale
```

```
## 10 10_Aa_fe_4d_con_2_1         0.3673656 38.98051 1.345867 0.5900473 2-Fem
ale
## 11 11_Aa_fe_4d_con_3_1         0.3928250 39.99997 1.449084 0.6014124 2-Fem
ale
## 12 12_Aa_fe_4d_con_4_1         0.3355749 37.99977 1.220683 0.5801670 2-Fem
ale
## 13 13_Aa_fe_4d_ira_1_1         0.4402727 40.99953 1.634984 0.7131874 2-Fem
ale
## 14 14_Aa_fe_4d_ira_2_1         0.3687104 35.00000 1.310894 0.5896714 2-Fem
ale
## 15 15_Aa_fe_4d_ira_3_1         0.4260584 35.99088 1.526788 0.6613638 2-Fem
ale
## 16 16_Aa_fe_4d_ira_4_1         0.3784201 35.99941 1.356076 0.5908777 2-Fem
ale
## 17  17_Aa_fe_p_con_1_1         0.7142654 45.00000 2.718967 0.8632037 2-Fem
ale
## 18  18_Aa_fe_p_con_2_1         0.7601570 43.99933 2.876578 0.9196417 2-Fem
ale
## 19  19_Aa_fe_p_con_3_1         0.8551288 48.00000 3.310376 0.9403287 2-Fem
ale
## 20  20_Aa_fe_p_con_4_1         0.4447495 40.00000 1.640627 0.6648053 2-Fem
ale
## 21  21_Aa_fe_p_ira_1_1         0.8307195 48.00000 3.215882 0.9223384 2-Fem
ale
## 22  22_Aa_fe_p_ira_2_1         0.8364856 49.00000 3.255452 0.9237309 2-Fem
ale
## 23  23_Aa_fe_p_ira_3_1         0.6352701 44.00000 2.403982 0.8377042 2-Fem
ale
## 24  24_Aa_fe_p_ira_4_1         0.5733687 40.00000 2.115088 0.8018672 2-Fem
ale
## 25  25_Aa_m_1d_con_1_1         0.5840240 50.99996 2.296281 0.7985648   1-M
ale
## 26  26_Aa_m_1d_con_2_1         0.7406619 53.00000 2.940644 0.9076571   1-M
ale
## 27  27_Aa_m_1d_con_3_1         0.6364175 49.99904 2.489680 0.8699462   1-M
ale
## 28  28_Aa_m_1d_con_4_1         0.5513585 45.99977 2.110954 0.8176010   1-M
ale
## 29  29_Aa_m_1d_ira_1_1         0.5856204 44.99998 2.229259 0.8436617   1-M
ale
## 30  30_Aa_m_1d_ira_2_1         0.6167944 44.99993 2.347928 0.8627697   1-M
ale
## 31  31_Aa_m_1d_ira_3_1         0.5450621 36.88833 1.968174 0.8008341   1-M
ale
## 32  32_Aa_m_1d_ira_4_1         0.5663660 39.99991 2.089256 0.8349982   1-M
ale
## 33  33_Aa_m_4d_con_1_1         0.3926679 37.99469 1.428363 0.6560487   1-M
ale
## 34  34_Aa_m_4d_con_2_1         0.4270078 30.77900 1.466339 0.6956308   1-M
ale
```

```
## 35  35_Aa_m_4d_con_3_1         0.3902456 27.99779 1.300378 0.6306110   1-M
ale
## 36  36_Aa_m_4d_con_4_1         0.4368995 25.99604 1.423461 0.6537783   1-M
ale
## 37  37_Aa_m_4d_ira_1_1         0.4204578 28.90147 1.415806 0.6417680   1-M
ale
## 38  38_Aa_m_4d_ira_2_1         0.3947282 24.99997 1.270581 0.6073143   1-M
ale
## 39  39_Aa_m_4d_ira_3_1         0.3852395 33.99144 1.358493 0.6072802   1-M
ale
## 40  40_Aa_m_4d_ira_4_1         0.3421446 30.94494 1.174920 0.5868999   1-M
ale
## 41   41_Aa_m_p_con_1_1         0.7487692 49.00000 2.914075 0.9199040   1-M
ale
## 42   42_Aa_m_p_con_2_1         0.8087923 47.00000 3.113970 0.9381943   1-M
ale
## 43   43_Aa_m_p_con_3_1         0.7356088 56.00000 2.961084 0.9181881   1-M
ale
## 44   44_Aa_m_p_con_4_1         0.5452530 45.00000 2.075594 0.7681446   1-M
ale
## 45   45_Aa_m_p_ira_1_1         0.6588400 52.00000 2.603238 0.8338246   1-M
ale
## 46   46_Aa_m_p_ira_2_1         0.7890739 54.00000 3.147603 0.9362312   1-M
ale
## 47   47_Aa_m_p_ira_3_1         0.6124385 55.00000 2.454245 0.8615585   1-M
ale
## 48   48_Aa_m_p_ira_4_1         0.6611672 50.00000 2.586501 0.8443385   1-M
ale
##          Age1 Stage   Treatment1 SampleID Age_Irradiation Sample Treatment
Age
## 1  2-1d Adult Adult    2-Control        1     1_F_Control   F1DC         2
2
## 2  2-1d Adult Adult    2-Control        1     1_F_Control   F1DC         2
2
## 3  2-1d Adult Adult    2-Control        1     1_F_Control   F1DC         2
2
## 4  2-1d Adult Adult    2-Control        1     1_F_Control   F1DC         2
2
## 5  2-1d Adult Adult 1-Irradiated        2  1_F_Irradiated   F1DI         1
2
## 6  2-1d Adult Adult 1-Irradiated        2  1_F_Irradiated   F1DI         1
2
## 7  2-1d Adult Adult 1-Irradiated        2  1_F_Irradiated   F1DI         1
2
## 8  2-1d Adult Adult 1-Irradiated        2  1_F_Irradiated   F1DI         1
2
## 9  3-4d Adult Adult    2-Control        3     4_F_Control   F4DC         2
3
## 10 3-4d Adult Adult    2-Control        3     4_F_Control   F4DC         2
3
```

```
## 11 3-4d Adult Adult    2-Control        3     4_F_Control   F4DC          2
3
## 12 3-4d Adult Adult    2-Control        3     4_F_Control   F4DC          2
3
## 13 3-4d Adult Adult 1-Irradiated        4  4_F_Irradiated   F4DI          1
3
## 14 3-4d Adult Adult 1-Irradiated        4  4_F_Irradiated   F4DI          1
3
## 15 3-4d Adult Adult 1-Irradiated        4  4_F_Irradiated   F4DI          1
3
## 16 3-4d Adult Adult 1-Irradiated        4  4_F_Irradiated   F4DI          1
3
## 17     1-Pupa  Pupa    2-Control        5     0_F_Control    FPC          2
1
## 18     1-Pupa  Pupa    2-Control        5     0_F_Control    FPC          2
1
## 19     1-Pupa  Pupa    2-Control        5     0_F_Control    FPC          2
1
## 20     1-Pupa  Pupa    2-Control        5     0_F_Control    FPC          2
1
## 21     1-Pupa  Pupa 1-Irradiated        6  0_F_Irradiated    FPI          1
1
## 22     1-Pupa  Pupa 1-Irradiated        6  0_F_Irradiated    FPI          1
1
## 23     1-Pupa  Pupa 1-Irradiated        6  0_F_Irradiated    FPI          1
1
## 24     1-Pupa  Pupa 1-Irradiated        6  0_F_Irradiated    FPI          1
1
## 25 2-1d Adult Adult    2-Control        7     0_M_Control   M1DC          2
2
## 26 2-1d Adult Adult    2-Control        7     1_M_Control   M1DC          2
2
## 27 2-1d Adult Adult    2-Control        7     1_M_Control   M1DC          2
2
## 28 2-1d Adult Adult    2-Control        7     1_M_Control   M1DC          2
2
## 29 2-1d Adult Adult 1-Irradiated        8  1_M_Irradiated   M1DI          1
2
## 30 2-1d Adult Adult 1-Irradiated        8  1_M_Irradiated   M1DI          1
2
## 31 2-1d Adult Adult 1-Irradiated        8  1_M_Irradiated   M1DI          1
2
## 32 2-1d Adult Adult 1-Irradiated        8  1_M_Irradiated   M1DI          1
2
## 33 3-4d Adult Adult    2-Control        9     4_M_Control   M4DC          2
3
## 34 3-4d Adult Adult    2-Control        9     4_M_Control   M4DC          2
3
## 35 3-4d Adult Adult    2-Control        9     4_M_Control   M4DC          2
3
```

```
## 36 3-4d Adult Adult    2-Control         9     4_M_Control   M4DC          2
3
## 37 3-4d Adult Adult 1-Irradiated       10  4_M_Irradiated   M4DI          1
3
## 38 3-4d Adult Adult 1-Irradiated       10  4_M_Irradiated   M4DI          1
3
## 39 3-4d Adult Adult 1-Irradiated       10  4_M_Irradiated   M4DI          1
3
## 40 3-4d Adult Adult 1-Irradiated       10  4_M_Irradiated   M4DI          1
3
## 41     1-Pupa  Pupa    2-Control       11     0_M_Control    MPC          2
1
## 42     1-Pupa  Pupa    2-Control       11     0_M_Control    MPC          2
1
## 43     1-Pupa  Pupa    2-Control       11     0_M_Control    MPC          2
1
## 44     1-Pupa  Pupa    2-Control       11     0_M_Control    MPC          2
1
## 45     1-Pupa  Pupa 1-Irradiated       12  0_M_Irradiated    MPI          1
1
## 46     1-Pupa  Pupa 1-Irradiated       12  0_M_Irradiated    MPI          1
1
## 47     1-Pupa  Pupa 1-Irradiated       12  0_M_Irradiated    MPI          1
1
## 48     1-Pupa  Pupa 1-Irradiated       12  0_M_Irradiated    MPI          1
1
##    Sex
## 1    2
## 2    2
## 3    2
## 4    2
## 5    2
## 6    2
## 7    2
## 8    2
## 9    2
## 10   2
## 11   2
## 12   2
## 13   2
## 14   2
## 15   2
## 16   2
## 17   2
## 18   2
## 19   2
## 20   2
## 21   2
## 22   2
## 23   2
```

```
## 24   2
## 25   1
## 26   1
## 27   1
## 28   1
## 29   1
## 30   1
## 31   1
## 32   1
## 33   1
## 34   1
## 35   1
## 36   1
## 37   1
## 38   1
## 39   1
## 40   1
## 41   1
## 42   1
## 43   1
## 44   1
## 45   1
## 46   1
## 47   1
## 48   1
```

```
library(ggplot2)
```

```
## Warning: package 'ggplot2' was built under R version 4.0.5
```

```
library(lattice)
library(gcookbook)
library(datasets)
library(MASS)
library(survival)
library(rmarkdown)
library(knitr)
library(coxme)
```

```
## Loading required package: bdsmatrix
```

```
##
## Attaching package: 'bdsmatrix'
```

```
## The following object is masked from 'package:base':
##
##     backsolve
```

```
library(lme4)
```

```
## Loading required package: Matrix
```

```r
library(nlme)

##
## Attaching package: 'nlme'

## The following object is masked from 'package:lme4':
##
##     lmList

library(gapminder)
library(rcompanion)
library(FSA)

## ## FSA v0.8.32. See citation('FSA') if used in publication.
## ## Run fishR() for related website and fishR('IFAR') for related book.

library(ggthemes) # Load

## Warning: package 'ggthemes' was built under R version 4.0.5
```

Statiscic for the manuscript (in sequence)

```r
wilcox.test(Richness~Treatment1, data=alph)

## Warning in wilcox.test.default(x = c(28.97187469, 50, 47, 45.99999011,
## 40.99953068, : cannot compute exact p-value with ties

##
##  Wilcoxon rank sum test with continuity correction
##
## data:  Richness by Treatment1
## W = 296.5, p-value = 0.869
## alternative hypothesis: true location shift is not equal to 0

wilcox.test(Richness~Sex1, data=alph)

## Warning in wilcox.test.default(x = c(50.99996411, 52.99999994, 49.99904148
, :
## cannot compute exact p-value with ties

##
##  Wilcoxon rank sum test with continuity correction
##
## data:  Richness by Sex1
## W = 335, p-value = 0.3376
## alternative hypothesis: true location shift is not equal to 0

kruskal.test(Richness~Age1, data = alph)

##
##  Kruskal-Wallis rank sum test
##
## data:  Richness by Age1
## Kruskal-Wallis chi-squared = 23.451, df = 2, p-value = 8.085e-06
```

```
tmp <- dunnTest(Richness~Age1, data = alph, method ="none")

## Warning: Age1 was coerced to a factor.

tmp

## Dunn (1964) Kruskal-Wallis multiple comparison

##   with no adjustment for p-values.

##                 Comparison        Z      P.unadj        P.adj
## 1     1-Pupa - 2-1d Adult 1.982586 4.741372e-02 4.741372e-02
## 2     1-Pupa - 3-4d Adult 4.817557 1.453264e-06 1.453264e-06
## 3 2-1d Adult - 3-4d Adult 2.834971 4.582979e-03 4.582979e-03

kruskal.test(Pielou.s.evenness~Sample, data = alph)

##
##  Kruskal-Wallis rank sum test
##
## data:  Pielou.s.evenness by Sample
## Kruskal-Wallis chi-squared = 37.347, df = 11, p-value = 0.0001008

tmp <- dunnTest(Pielou.s.evenness~Sample, data = alph)

## Warning: Sample was coerced to a factor.

tmp

## Dunn (1964) Kruskal-Wallis multiple comparison

##   p-values adjusted with the Holm method.

##     Comparison           Z     P.unadj      P.adj
## 1  F1DC - F1DI -1.38895975 0.164844990 1.00000000
## 2  F1DC - F4DC  1.36370594 0.172660135 1.00000000
## 3  F1DI - F4DC  2.75266568 0.005911223 0.32511725
## 4  F1DC - F4DI  1.01015254 0.312422211 1.00000000
## 5  F1DI - F4DI  2.39911229 0.016434874 0.75600419
## 6  F4DC - F4DI -0.35355339 0.723673610 1.00000000
## 7   F1DC - FPC -1.69200551 0.090644923 1.00000000
## 8   F1DI - FPC -0.30304576 0.761854986 1.00000000
## 9   F4DC - FPC -3.05571145 0.002245272 0.14145215
## 10  F4DI - FPC -2.70215806 0.006889100 0.37201143
## 11  F1DC - FPI -1.91928983 0.054947664 1.00000000
## 12  F1DI - FPI -0.53033009 0.595883091 1.00000000
## 13  F4DC - FPI -3.28299577 0.001027102 0.06778874
## 14  F4DI - FPI -2.92944238 0.003395708 0.20374246
## 15   FPC - FPI -0.22728432 0.820202663 1.00000000
## 16 F1DC - M1DC -1.23743687 0.215924939 1.00000000
## 17 F1DI - M1DC  0.15152288 0.879563262 1.00000000
## 18 F4DC - M1DC -2.60114280 0.009291377 0.47386022
## 19 F4DI - M1DC -2.24758941 0.024602383 1.00000000
```

```
## 20  FPC - M1DC  0.45456865 0.649419597 1.00000000
## 21  FPI - M1DC  0.68185297 0.495331928 1.00000000
## 22 F1DC - M1DI -0.80812204 0.419020334 1.00000000
## 23 F1DI - M1DI  0.58083771 0.561349835 1.00000000
## 24 F4DC - M1DI -2.17182797 0.029868641 1.00000000
## 25 F4DI - M1DI -1.81827458 0.069022176 1.00000000
## 26  FPC - M1DI  0.88388348 0.376759118 1.00000000
## 27  FPI - M1DI  1.11116780 0.266496129 1.00000000
## 28 M1DC - M1DI  0.42931483 0.667694125 1.00000000
## 29 F1DC - M4DC  0.85862966 0.390544867 1.00000000
## 30 F1DI - M4DC  2.24758941 0.024602383 1.00000000
## 31 F4DC - M4DC -0.50507627 0.613505266 1.00000000
## 32 F4DI - M4DC -0.15152288 0.879563262 1.00000000
## 33  FPC - M4DC  2.55063517 0.010752682 0.52688140
## 34  FPI - M4DC  2.77791950 0.005470817 0.31183657
## 35 M1DC - M4DC  2.09606653 0.036076290 1.00000000
## 36 M1DI - M4DC  1.66675170 0.095563788 1.00000000
## 37 F1DC - M4DI  1.21218305 0.225442317 1.00000000
## 38 F1DI - M4DI  2.60114280 0.009291377 0.46456884
## 39 F4DC - M4DI -0.15152288 0.879563262 1.00000000
## 40 F4DI - M4DI  0.20203051 0.839892873 1.00000000
## 41  FPC - M4DI  2.90418857 0.003682063 0.21724173
## 42  FPI - M4DI  3.13147289 0.001739318 0.11305570
## 43 M1DC - M4DI  2.44961992 0.014300708 0.67213328
## 44 M1DI - M4DI  2.02030509 0.043351751 1.00000000
## 45 M4DC - M4DI  0.35355339 0.723673610 1.00000000
## 46  F1DC - MPC -1.76776695 0.077099872 1.00000000
## 47  F1DI - MPC -0.37880720 0.704831037 1.00000000
## 48  F4DC - MPC -3.13147289 0.001739318 0.11131638
## 49  F4DI - MPC -2.77791950 0.005470817 0.30636575
## 50   FPC - MPC -0.07576144 0.939608894 1.00000000
## 51   FPI - MPC  0.15152288 0.879563262 1.00000000
## 52  M1DC - MPC -0.53033009 0.595883091 1.00000000
## 53  M1DI - MPC -0.95964492 0.337233954 1.00000000
## 54  M4DC - MPC -2.62639662 0.008629420 0.44872985
## 55  M4DI - MPC -2.97995001 0.002882954 0.17586021
## 56  F1DC - MPI -1.69200551 0.090644923 1.00000000
## 57  F1DI - MPI -0.30304576 0.761854986 1.00000000
## 58  F4DC - MPI -3.05571145 0.002245272 0.13920688
## 59  F4DI - MPI -2.70215806 0.006889100 0.36512233
## 60   FPC - MPI  0.00000000 1.000000000 1.00000000
## 61   FPI - MPI  0.22728432 0.820202663 1.00000000
## 62  M1DC - MPI -0.45456865 0.649419597 1.00000000
## 63  M1DI - MPI -0.88388348 0.376759118 1.00000000
## 64  M4DC - MPI -2.55063517 0.010752682 0.51612872
## 65  M4DI - MPI -2.90418857 0.003682063 0.21355967
## 66   MPC - MPI  0.07576144 0.939608894 1.00000000

kruskal.test(Shannon~Sample, data = alph)
```

```
##
##  Kruskal-Wallis rank sum test
##
## data:  Shannon by Sample
## Kruskal-Wallis chi-squared = 37.39, df = 11, p-value = 9.91e-05
```

```r
tmp <- dunnTest(Shannon~Sample, data = alph, method ="none")
```

```
## Warning: Sample was coerced to a factor.
```

```r
tmp
```

```
## Dunn (1964) Kruskal-Wallis multiple comparison
```

```
##   with no adjustment for p-values.
```

```
##     Comparison           Z     P.unadj       P.adj
## 1  F1DC - F1DI -1.21218305 0.225442317 0.225442317
## 2  F1DC - F4DC  1.18692924 0.235255533 0.235255533
## 3  F1DI - F4DC  2.39911229 0.016434874 0.016434874
## 4  F1DC - F4DI  0.93439110 0.350102185 0.350102185
## 5  F1DI - F4DI  2.14657416 0.031827199 0.031827199
## 6  F4DC - F4DI -0.25253814 0.800625141 0.800625141
## 7   F1DC - FPC -1.56573644 0.117410331 0.117410331
## 8   F1DI - FPC -0.35355339 0.723673610 0.723673610
## 9   F4DC - FPC -2.75266568 0.005911223 0.005911223
## 10  F4DI - FPC -2.50012755 0.012414860 0.012414860
## 11  F1DC - FPI -1.81827458 0.069022176 0.069022176
## 12  F1DI - FPI -0.60609153 0.544453977 0.544453977
## 13  F4DC - FPI -3.00520382 0.002654029 0.002654029
## 14  F4DI - FPI -2.75266568 0.005911223 0.005911223
## 15   FPC - FPI -0.25253814 0.800625141 0.800625141
## 16 F1DC - M1DC -1.28794449 0.197765285 0.197765285
## 17 F1DI - M1DC -0.07576144 0.939608894 0.939608894
## 18 F4DC - M1DC -2.47487373 0.013328329 0.013328329
## 19 F4DI - M1DC -2.22233560 0.026260634 0.026260634
## 20  FPC - M1DC  0.27779195 0.781172071 0.781172071
## 21  FPI - M1DC  0.53033009 0.595883091 0.595883091
## 22 F1DC - M1DI -0.68185297 0.495331928 0.495331928
## 23 F1DI - M1DI  0.53033009 0.595883091 0.595883091
## 24 F4DC - M1DI -1.86878221 0.061653121 0.061653121
## 25 F4DI - M1DI -1.61624407 0.106041554 0.106041554
## 26  FPC - M1DI  0.88388348 0.376759118 0.376759118
## 27  FPI - M1DI  1.13642161 0.255780155 0.255780155
## 28 M1DC - M1DI  0.60609153 0.544453977 0.544453977
## 29 F1DC - M4DC  1.03540636 0.300479178 0.300479178
## 30 F1DI - M4DC  2.24758941 0.024602383 0.024602383
## 31 F4DC - M4DC -0.15152288 0.879563262 0.879563262
## 32 F4DI - M4DC  0.10101525 0.919538351 0.919538351
## 33  FPC - M4DC  2.60114280 0.009291377 0.009291377
## 34  FPI - M4DC  2.85368094 0.004321591 0.004321591
```

```
## 35 M1DC - M4DC  2.32335085 0.020160312 0.020160312
## 36 M1DI - M4DC  1.71725933 0.085931800 0.085931800
## 37 F1DC - M4DI  1.48997500 0.136230809 0.136230809
## 38 F1DI - M4DI  2.70215806 0.006889100 0.006889100
## 39 F4DC - M4DI  0.30304576 0.761854986 0.761854986
## 40 F4DI - M4DI  0.55558390 0.578495341 0.578495341
## 41  FPC - M4DI  3.05571145 0.002245272 0.002245272
## 42  FPI - M4DI  3.30824958 0.000938811 0.000938811
## 43 M1DC - M4DI  2.77791950 0.005470817 0.005470817
## 44 M1DI - M4DI  2.17182797 0.029868641 0.029868641
## 45 M4DC - M4DI  0.45456865 0.649419597 0.649419597
## 46  F1DC - MPC -1.76776695 0.077099872 0.077099872
## 47  F1DI - MPC -0.55558390 0.578495341 0.578495341
## 48  F4DC - MPC -2.95469619 0.003129771 0.003129771
## 49  F4DI - MPC -2.70215806 0.006889100 0.006889100
## 50   FPC - MPC -0.20203051 0.839892873 0.839892873
## 51   FPI - MPC  0.05050763 0.959717872 0.959717872
## 52  M1DC - MPC -0.47982246 0.631353642 0.631353642
## 53  M1DI - MPC -1.08591399 0.277517049 0.277517049
## 54  M4DC - MPC -2.80317331 0.005060247 0.005060247
## 55  M4DI - MPC -3.25774196 0.001123025 0.001123025
## 56  F1DC - MPI -1.76776695 0.077099872 0.077099872
## 57  F1DI - MPI -0.55558390 0.578495341 0.578495341
## 58  F4DC - MPI -2.95469619 0.003129771 0.003129771
## 59  F4DI - MPI -2.70215806 0.006889100 0.006889100
## 60   FPC - MPI -0.20203051 0.839892873 0.839892873
## 61   FPI - MPI  0.05050763 0.959717872 0.959717872
## 62  M1DC - MPI -0.47982246 0.631353642 0.631353642
## 63  M1DI - MPI -1.08591399 0.277517049 0.277517049
## 64  M4DC - MPI -2.80317331 0.005060247 0.005060247
## 65  M4DI - MPI -3.25774196 0.001123025 0.001123025
## 66   MPC - MPI  0.00000000 1.000000000 1.000000000
```

```
kruskal.test(Simpson~Sample, data = alph)
```

```
##
##  Kruskal-Wallis rank sum test
##
## data:  Simpson by Sample
## Kruskal-Wallis chi-squared = 35.737, df = 11, p-value = 0.0001871
```

```
tmp <- dunnTest(Simpson~Sample, data = alph)
```

```
## Warning: Sample was coerced to a factor.
```

```
tmp
```

```
## Dunn (1964) Kruskal-Wallis multiple comparison
```

```
##   p-values adjusted with the Holm method.
```

```
##     Comparison           Z     P.unadj      P.adj
## 1  F1DC - F1DI -1.28794449 0.197765285 1.00000000
## 2  F1DC - F4DC  1.56573644 0.117410331 1.00000000
## 3  F1DI - F4DC  2.85368094 0.004321591 0.25497387
## 4  F1DC - F4DI  1.08591399 0.277517049 1.00000000
## 5  F1DI - F4DI  2.37385848 0.017603296 0.84495821
## 6  F4DC - F4DI -0.47982246 0.631353642 1.00000000
## 7   F1DC - FPC -1.48997500 0.136230809 1.00000000
## 8   F1DI - FPC -0.20203051 0.839892873 1.00000000
## 9   F4DC - FPC -3.05571145 0.002245272 0.13920688
## 10  F4DI - FPC -2.57588899 0.009998274 0.51991025
## 11  F1DC - FPI -1.56573644 0.117410331 1.00000000
## 12  F1DI - FPI -0.27779195 0.781172071 1.00000000
## 13  F4DC - FPI -3.13147289 0.001739318 0.11305570
## 14  F4DI - FPI -2.65165043 0.008009942 0.44855677
## 15   FPC - FPI -0.07576144 0.939608894 1.00000000
## 16 F1DC - M1DC -1.08591399 0.277517049 1.00000000
## 17 F1DI - M1DC  0.20203051 0.839892873 1.00000000
## 18 F4DC - M1DC -2.65165043 0.008009942 0.44054683
## 19 F4DI - M1DC -2.17182797 0.029868641 1.00000000
## 20  FPC - M1DC  0.40406102 0.686167850 1.00000000
## 21  FPI - M1DC  0.47982246 0.631353642 1.00000000
## 22 F1DC - M1DI -0.98489873 0.324673817 1.00000000
## 23 F1DI - M1DI  0.30304576 0.761854986 1.00000000
## 24 F4DC - M1DI -2.55063517 0.010752682 0.54838677
## 25 F4DI - M1DI -2.07081272 0.038376301 1.00000000
## 26  FPC - M1DI  0.50507627 0.613505266 1.00000000
## 27  FPI - M1DI  0.58083771 0.561349835 1.00000000
## 28 M1DC - M1DI  0.10101525 0.919538351 1.00000000
## 29 F1DC - M4DC  0.78286822 0.433704500 1.00000000
## 30 F1DI - M4DC  2.07081272 0.038376301 1.00000000
## 31 F4DC - M4DC -0.78286822 0.433704500 1.00000000
## 32 F4DI - M4DC -0.30304576 0.761854986 1.00000000
## 33  FPC - M4DC  2.27284323 0.023035629 1.00000000
## 34  FPI - M4DC  2.34860467 0.018843902 0.88566340
## 35 M1DC - M4DC  1.86878221 0.061653121 1.00000000
## 36 M1DI - M4DC  1.76776695 0.077099872 1.00000000
## 37 F1DC - M4DI  1.36370594 0.172660135 1.00000000
## 38 F1DI - M4DI  2.65165043 0.008009942 0.43253689
## 39 F4DC - M4DI -0.20203051 0.839892873 1.00000000
## 40 F4DI - M4DI  0.27779195 0.781172071 1.00000000
## 41  FPC - M4DI  2.85368094 0.004321591 0.25065228
## 42  FPI - M4DI  2.92944238 0.003395708 0.20713817
## 43 M1DC - M4DI  2.44961992 0.014300708 0.70073469
## 44 M1DI - M4DI  2.34860467 0.018843902 0.86681950
## 45 M4DC - M4DI  0.58083771 0.561349835 1.00000000
## 46  F1DC - MPC -1.71725933 0.085931800 1.00000000
## 47  F1DI - MPC -0.42931483 0.667694125 1.00000000
## 48  F4DC - MPC -3.28299577 0.001027102 0.06778874
## 49  F4DI - MPC -2.80317331 0.005060247 0.28843407
```

```
## 50   FPC - MPC -0.22728432 0.820202663 1.00000000
## 51   FPI - MPC -0.15152288 0.879563262 1.00000000
## 52  M1DC - MPC -0.63134534 0.527814747 1.00000000
## 53  M1DI - MPC -0.73236059 0.463948504 1.00000000
## 54  M4DC - MPC -2.50012755 0.012414860 0.62074300
## 55  M4DI - MPC -3.08096526 0.002063307 0.13205166
## 56  F1DC - MPI -1.51522882 0.129714468 1.00000000
## 57  F1DI - MPI -0.22728432 0.820202663 1.00000000
## 58  F4DC - MPI -3.08096526 0.002063307 0.12998835
## 59  F4DI - MPI -2.60114280 0.009291377 0.49244297
## 60   FPC - MPI -0.02525381 0.979852514 0.97985251
## 61   FPI - MPI  0.05050763 0.959717872 1.00000000
## 62  M1DC - MPI -0.42931483 0.667694125 1.00000000
## 63  M1DI - MPI -0.53033009 0.595883091 1.00000000
## 64  M4DC - MPI -2.29809704 0.021556267 0.97003200
## 65  M4DI - MPI -2.87893475 0.003990209 0.23941253
## 66   MPC - MPI  0.20203051 0.839892873 1.00000000
```

```r
kruskal.test(Shannon~Age1, data = alph)
```

```
## 
##  Kruskal-Wallis rank sum test
## 
## data:  Shannon by Age1
## Kruskal-Wallis chi-squared = 34.839, df = 2, p-value = 2.721e-08
```

```r
tmp <- dunnTest(Shannon~Age1, data = alph, method ="none")
```

```
## Warning: Age1 was coerced to a factor.
```

```r
tmp
```

```
## Dunn (1964) Kruskal-Wallis multiple comparison
```

```
##   with no adjustment for p-values.
```

```
##                 Comparison        Z      P.unadj        P.adj
## 1     1-Pupa - 2-1d Adult 1.868782 6.165312e-02 6.165312e-02
## 2     1-Pupa - 3-4d Adult 5.783123 7.332627e-09 7.332627e-09
## 3 2-1d Adult - 3-4d Adult 3.914341 9.065141e-05 9.065141e-05
```

```r
wilcox.test(Shannon~Treatment1, data=alph)
```

```
## 
##  Wilcoxon rank sum exact test
## 
## data:  Shannon by Treatment1
## W = 301, p-value = 0.7984
## alternative hypothesis: true location shift is not equal to 0
```

```r
wilcox.test(Shannon~Sex1, data=alph)
```

```
##
##  Wilcoxon rank sum exact test
##
## data:  Shannon by Sex1
## W = 298, p-value = 0.8461
## alternative hypothesis: true location shift is not equal to 0
```

Statisctics for preparing table 1

```
#Pielou.s.evenness
kruskal.test(Pielou.s.evenness~Sample, data = alph)

##
##  Kruskal-Wallis rank sum test
##
## data:  Pielou.s.evenness by Sample
## Kruskal-Wallis chi-squared = 37.347, df = 11, p-value = 0.0001008

tmp <- dunnTest(Pielou.s.evenness~Sample, data = alph, method ="none")

## Warning: Sample was coerced to a factor.

tmp

## Dunn (1964) Kruskal-Wallis multiple comparison

##   with no adjustment for p-values.

##     Comparison           Z      P.unadj       P.adj
## 1  F1DC - F1DI -1.38895975 0.164844990 0.164844990
## 2  F1DC - F4DC  1.36370594 0.172660135 0.172660135
## 3  F1DI - F4DC  2.75266568 0.005911223 0.005911223
## 4  F1DC - F4DI  1.01015254 0.312422211 0.312422211
## 5  F1DI - F4DI  2.39911229 0.016434874 0.016434874
## 6  F4DC - F4DI -0.35355339 0.723673610 0.723673610
## 7   F1DC - FPC -1.69200551 0.090644923 0.090644923
## 8   F1DI - FPC -0.30304576 0.761854986 0.761854986
## 9   F4DC - FPC -3.05571145 0.002245272 0.002245272
## 10  F4DI - FPC -2.70215806 0.006889100 0.006889100
## 11  F1DC - FPI -1.91928983 0.054947664 0.054947664
## 12  F1DI - FPI -0.53033009 0.595883091 0.595883091
## 13  F4DC - FPI -3.28299577 0.001027102 0.001027102
## 14  F4DI - FPI -2.92944238 0.003395708 0.003395708
## 15   FPC - FPI -0.22728432 0.820202663 0.820202663
## 16 F1DC - M1DC -1.23743687 0.215924939 0.215924939
## 17 F1DI - M1DC  0.15152288 0.879563262 0.879563262
## 18 F4DC - M1DC -2.60114280 0.009291377 0.009291377
## 19 F4DI - M1DC -2.24758941 0.024602383 0.024602383
## 20  FPC - M1DC  0.45456865 0.649419597 0.649419597
## 21  FPI - M1DC  0.68185297 0.495331928 0.495331928
## 22 F1DC - M1DI -0.80812204 0.419020334 0.419020334
## 23 F1DI - M1DI  0.58083771 0.561349835 0.561349835
```

```
## 24 F4DC - M1DI -2.17182797 0.029868641 0.029868641
## 25 F4DI - M1DI -1.81827458 0.069022176 0.069022176
## 26  FPC - M1DI  0.88388348 0.376759118 0.376759118
## 27  FPI - M1DI  1.11116780 0.266496129 0.266496129
## 28 M1DC - M1DI  0.42931483 0.667694125 0.667694125
## 29 F1DC - M4DC  0.85862966 0.390544867 0.390544867
## 30 F1DI - M4DC  2.24758941 0.024602383 0.024602383
## 31 F4DC - M4DC -0.50507627 0.613505266 0.613505266
## 32 F4DI - M4DC -0.15152288 0.879563262 0.879563262
## 33  FPC - M4DC  2.55063517 0.010752682 0.010752682
## 34  FPI - M4DC  2.77791950 0.005470817 0.005470817
## 35 M1DC - M4DC  2.09606653 0.036076290 0.036076290
## 36 M1DI - M4DC  1.66675170 0.095563788 0.095563788
## 37 F1DC - M4DI  1.21218305 0.225442317 0.225442317
## 38 F1DI - M4DI  2.60114280 0.009291377 0.009291377
## 39 F4DC - M4DI -0.15152288 0.879563262 0.879563262
## 40 F4DI - M4DI  0.20203051 0.839892873 0.839892873
## 41  FPC - M4DI  2.90418857 0.003682063 0.003682063
## 42  FPI - M4DI  3.13147289 0.001739318 0.001739318
## 43 M1DC - M4DI  2.44961992 0.014300708 0.014300708
## 44 M1DI - M4DI  2.02030509 0.043351751 0.043351751
## 45 M4DC - M4DI  0.35355339 0.723673610 0.723673610
## 46  F1DC - MPC -1.76776695 0.077099872 0.077099872
## 47  F1DI - MPC -0.37880720 0.704831037 0.704831037
## 48  F4DC - MPC -3.13147289 0.001739318 0.001739318
## 49  F4DI - MPC -2.77791950 0.005470817 0.005470817
## 50   FPC - MPC -0.07576144 0.939608894 0.939608894
## 51   FPI - MPC  0.15152288 0.879563262 0.879563262
## 52  M1DC - MPC -0.53033009 0.595883091 0.595883091
## 53  M1DI - MPC -0.95964492 0.337233954 0.337233954
## 54  M4DC - MPC -2.62639662 0.008629420 0.008629420
## 55  M4DI - MPC -2.97995001 0.002882954 0.002882954
## 56  F1DC - MPI -1.69200551 0.090644923 0.090644923
## 57  F1DI - MPI -0.30304576 0.761854986 0.761854986
## 58  F4DC - MPI -3.05571145 0.002245272 0.002245272
## 59  F4DI - MPI -2.70215806 0.006889100 0.006889100
## 60   FPC - MPI  0.00000000 1.000000000 1.000000000
## 61   FPI - MPI  0.22728432 0.820202663 0.820202663
## 62  M1DC - MPI -0.45456865 0.649419597 0.649419597
## 63  M1DI - MPI -0.88388348 0.376759118 0.376759118
## 64  M4DC - MPI -2.55063517 0.010752682 0.010752682
## 65  M4DI - MPI -2.90418857 0.003682063 0.003682063
## 66   MPC - MPI  0.07576144 0.939608894 0.939608894

#Richness
kruskal.test(Richness~Sample, data = alph)

##
##  Kruskal-Wallis rank sum test
##
```

```
## data:  Richness by Sample
## Kruskal-Wallis chi-squared = 37.15, df = 11, p-value = 0.0001088

tmp <- dunnTest(Richness~Sample, data = alph, method ="none")

## Warning: Sample was coerced to a factor.

tmp

## Dunn (1964) Kruskal-Wallis multiple comparison
##   with no adjustment for p-values.

##      Comparison           Z      P.unadj        P.adj
## 1   F1DC - F1DI -1.73002709 0.0836254354 0.0836254354
## 2   F1DC - F4DC -0.70716436 0.4794643453 0.4794643453
## 3   F1DI - F4DC  1.02286273 0.3063727526 0.3063727526
## 4   F1DC - F4DI -0.35358218 0.7236520319 0.7236520319
## 5   F1DI - F4DI  1.37644491 0.1686839318 0.1686839318
## 6   F4DC - F4DI  0.35358218 0.7236520319 0.7236520319
## 7    F1DC - FPC -1.75528296 0.0792109198 0.0792109198
## 8    F1DI - FPC -0.02525587 0.9798508734 0.9798508734
## 9    F4DC - FPC -1.04811860 0.2945839670 0.2945839670
## 10   F4DI - FPC -1.40170078 0.1610046175 0.1610046175
## 11   F1DC - FPI -1.99521372 0.0460195749 0.0460195749
## 12   F1DI - FPI -0.26518663 0.7908656908 0.7908656908
## 13   F4DC - FPI -1.28804937 0.1977287795 0.1977287795
## 14   F4DI - FPI -1.64163154 0.1006663862 0.1006663862
## 15    FPC - FPI -0.23993076 0.8103839309 0.8103839309
## 16 F1DC - M1DC -2.85391330 0.0043184315 0.0043184315
## 17 F1DI - M1DC -1.12388621 0.2610613016 0.2610613016
## 18 F4DC - M1DC -2.14674894 0.0318132740 0.0318132740
## 19 F4DI - M1DC -2.50033112 0.0124077274 0.0124077274
## 20  FPC - M1DC -1.09863034 0.2719293377 0.2719293377
## 21  FPI - M1DC -0.85869958 0.3905062838 0.3905062838
## 22 F1DC - M1DI -1.13651415 0.2557414485 0.2557414485
## 23 F1DI - M1DI  0.59351294 0.5528379236 0.5528379236
## 24 F4DC - M1DI -0.42934979 0.6676686887 0.6676686887
## 25 F4DI - M1DI -0.78293197 0.4336670640 0.4336670640
## 26  FPC - M1DI  0.61876881 0.5360686702 0.5360686702
## 27  FPI - M1DI  0.85869958 0.3905062838 0.3905062838
## 28 M1DC - M1DI  1.71739915 0.0859062661 0.0859062661
## 29 F1DC - M4DC  0.42934979 0.6676686887 0.6676686887
## 30 F1DI - M4DC  2.15937688 0.0308209402 0.0308209402
## 31 F4DC - M4DC  1.13651415 0.2557414485 0.2557414485
## 32 F4DI - M4DC  0.78293197 0.4336670640 0.4336670640
## 33  FPC - M4DC  2.18463275 0.0289157848 0.0289157848
## 34  FPI - M4DC  2.42456351 0.0153268021 0.0153268021
## 35 M1DC - M4DC  3.28326309 0.0010261285 0.0010261285
## 36 M1DI - M4DC  1.56586393 0.1173804746 0.1173804746
## 37 F1DC - M4DI  0.58088501 0.5613179571 0.5613179571
## 38 F1DI - M4DI  2.31091210 0.0208377107 0.0208377107
```

```
## 39 F4DC - M4DI  1.28804937 0.1977287795 0.1977287795
## 40 F4DI - M4DI  0.93446719 0.3500629544 0.3500629544
## 41  FPC - M4DI  2.33616797 0.0194824891 0.0194824891
## 42  FPI - M4DI  2.57609873 0.0099922110 0.0099922110
## 43 M1DC - M4DI  3.43479831 0.0005929950 0.0005929950
## 44 M1DI - M4DI  1.71739915 0.0859062661 0.0859062661
## 45 M4DC - M4DI  0.15153522 0.8795535305 0.8795535305
## 46  F1DC - MPC -2.62661047 0.0086239994 0.0086239994
## 47  F1DI - MPC -0.89658338 0.3699412688 0.3699412688
## 48  F4DC - MPC -1.91944611 0.0549278997 0.0549278997
## 49  F4DI - MPC -2.27302829 0.0230244750 0.0230244750
## 50   FPC - MPC -0.87132751 0.3835753526 0.3835753526
## 51   FPI - MPC -0.63139675 0.5277811419 0.5277811419
## 52  M1DC - MPC  0.22730283 0.8201882733 0.8201882733
## 53  M1DI - MPC -1.49009633 0.1361989103 0.1361989103
## 54  M4DC - MPC -3.05596026 0.0022434099 0.0022434099
## 55  M4DI - MPC -3.20749548 0.0013389616 0.0013389616
## 56  F1DC - MPI -3.30851896 0.0009379083 0.0009379083
## 57  F1DI - MPI -1.57849187 0.1144526544 0.1144526544
## 58  F4DC - MPI -2.60135460 0.0092856418 0.0092856418
## 59  F4DI - MPI -2.95493678 0.0031273313 0.0031273313
## 60   FPC - MPI -1.55323600 0.1203667637 0.1203667637
## 61   FPI - MPI -1.31330524 0.1890801150 0.1890801150
## 62  M1DC - MPI -0.45460566 0.6493929632 0.6493929632
## 63  M1DI - MPI -2.17200481 0.0298552999 0.0298552999
## 64  M4DC - MPI -3.73786875 0.0001855868 0.0001855868
## 65  M4DI - MPI -3.88940397 0.0001004907 0.0001004907
## 66   MPC - MPI -0.68190849 0.4952968180 0.4952968180

#shannon
kruskal.test(Shannon~Sample, data = alph)

##
##  Kruskal-Wallis rank sum test
##
## data:  Shannon by Sample
## Kruskal-Wallis chi-squared = 37.39, df = 11, p-value = 9.91e-05

tmp <- dunnTest(Shannon~Sample, data = alph, method ="none")

## Warning: Sample was coerced to a factor.

tmp

## Dunn (1964) Kruskal-Wallis multiple comparison
##   with no adjustment for p-values.

##     Comparison           Z     P.unadj       P.adj
## 1  F1DC - F1DI -1.21218305 0.225442317 0.225442317
## 2  F1DC - F4DC  1.18692924 0.235255533 0.235255533
## 3  F1DI - F4DC  2.39911229 0.016434874 0.016434874
```

```
## 4  F1DC - F4DI  0.93439110 0.350102185 0.350102185
## 5  F1DI - F4DI  2.14657416 0.031827199 0.031827199
## 6  F4DC - F4DI -0.25253814 0.800625141 0.800625141
## 7   F1DC - FPC -1.56573644 0.117410331 0.117410331
## 8   F1DI - FPC -0.35355339 0.723673610 0.723673610
## 9   F4DC - FPC -2.75266568 0.005911223 0.005911223
## 10  F4DI - FPC -2.50012755 0.012414860 0.012414860
## 11  F1DC - FPI -1.81827458 0.069022176 0.069022176
## 12  F1DI - FPI -0.60609153 0.544453977 0.544453977
## 13  F4DC - FPI -3.00520382 0.002654029 0.002654029
## 14  F4DI - FPI -2.75266568 0.005911223 0.005911223
## 15   FPC - FPI -0.25253814 0.800625141 0.800625141
## 16 F1DC - M1DC -1.28794449 0.197765285 0.197765285
## 17 F1DI - M1DC -0.07576144 0.939608894 0.939608894
## 18 F4DC - M1DC -2.47487373 0.013328329 0.013328329
## 19 F4DI - M1DC -2.22233560 0.026260634 0.026260634
## 20  FPC - M1DC  0.27779195 0.781172071 0.781172071
## 21  FPI - M1DC  0.53033009 0.595883091 0.595883091
## 22 F1DC - M1DI -0.68185297 0.495331928 0.495331928
## 23 F1DI - M1DI  0.53033009 0.595883091 0.595883091
## 24 F4DC - M1DI -1.86878221 0.061653121 0.061653121
## 25 F4DI - M1DI -1.61624407 0.106041554 0.106041554
## 26  FPC - M1DI  0.88388348 0.376759118 0.376759118
## 27  FPI - M1DI  1.13642161 0.255780155 0.255780155
## 28 M1DC - M1DI  0.60609153 0.544453977 0.544453977
## 29 F1DC - M4DC  1.03540636 0.300479178 0.300479178
## 30 F1DI - M4DC  2.24758941 0.024602383 0.024602383
## 31 F4DC - M4DC -0.15152288 0.879563262 0.879563262
## 32 F4DI - M4DC  0.10101525 0.919538351 0.919538351
## 33  FPC - M4DC  2.60114280 0.009291377 0.009291377
## 34  FPI - M4DC  2.85368094 0.004321591 0.004321591
## 35 M1DC - M4DC  2.32335085 0.020160312 0.020160312
## 36 M1DI - M4DC  1.71725933 0.085931800 0.085931800
## 37 F1DC - M4DI  1.48997500 0.136230809 0.136230809
## 38 F1DI - M4DI  2.70215806 0.006889100 0.006889100
## 39 F4DC - M4DI  0.30304576 0.761854986 0.761854986
## 40 F4DI - M4DI  0.55558390 0.578495341 0.578495341
## 41  FPC - M4DI  3.05571145 0.002245272 0.002245272
## 42  FPI - M4DI  3.30824958 0.000938811 0.000938811
## 43 M1DC - M4DI  2.77791950 0.005470817 0.005470817
## 44 M1DI - M4DI  2.17182797 0.029868641 0.029868641
## 45 M4DC - M4DI  0.45456865 0.649419597 0.649419597
## 46  F1DC - MPC -1.76776695 0.077099872 0.077099872
## 47  F1DI - MPC -0.55558390 0.578495341 0.578495341
## 48  F4DC - MPC -2.95469619 0.003129771 0.003129771
## 49  F4DI - MPC -2.70215806 0.006889100 0.006889100
## 50   FPC - MPC -0.20203051 0.839892873 0.839892873
## 51   FPI - MPC  0.05050763 0.959717872 0.959717872
## 52  M1DC - MPC -0.47982246 0.631353642 0.631353642
## 53  M1DI - MPC -1.08591399 0.277517049 0.277517049
```

```
## 54  M4DC - MPC -2.80317331 0.005060247 0.005060247
## 55  M4DI - MPC -3.25774196 0.001123025 0.001123025
## 56  F1DC - MPI -1.76776695 0.077099872 0.077099872
## 57  F1DI - MPI -0.55558390 0.578495341 0.578495341
## 58  F4DC - MPI -2.95469619 0.003129771 0.003129771
## 59  F4DI - MPI -2.70215806 0.006889100 0.006889100
## 60   FPC - MPI -0.20203051 0.839892873 0.839892873
## 61   FPI - MPI  0.05050763 0.959717872 0.959717872
## 62  M1DC - MPI -0.47982246 0.631353642 0.631353642
## 63  M1DI - MPI -1.08591399 0.277517049 0.277517049
## 64  M4DC - MPI -2.80317331 0.005060247 0.005060247
## 65  M4DI - MPI -3.25774196 0.001123025 0.001123025
## 66   MPC - MPI  0.00000000 1.000000000 1.000000000

#Simpson
kruskal.test(Simpson~Sample, data = alph)

##
##  Kruskal-Wallis rank sum test
##
## data:  Simpson by Sample
## Kruskal-Wallis chi-squared = 35.737, df = 11, p-value = 0.0001871

tmp <- dunnTest(Simpson~Sample, data = alph, method ="none")

## Warning: Sample was coerced to a factor.

tmp

## Dunn (1964) Kruskal-Wallis multiple comparison
##   with no adjustment for p-values.

##     Comparison           Z      P.unadj        P.adj
## 1   F1DC - F1DI -1.28794449 0.197765285 0.197765285
## 2   F1DC - F4DC  1.56573644 0.117410331 0.117410331
## 3   F1DI - F4DC  2.85368094 0.004321591 0.004321591
## 4   F1DC - F4DI  1.08591399 0.277517049 0.277517049
## 5   F1DI - F4DI  2.37385848 0.017603296 0.017603296
## 6   F4DC - F4DI -0.47982246 0.631353642 0.631353642
## 7    F1DC - FPC -1.48997500 0.136230809 0.136230809
## 8    F1DI - FPC -0.20203051 0.839892873 0.839892873
## 9    F4DC - FPC -3.05571145 0.002245272 0.002245272
## 10   F4DI - FPC -2.57588899 0.009998274 0.009998274
## 11   F1DC - FPI -1.56573644 0.117410331 0.117410331
## 12   F1DI - FPI -0.27779195 0.781172071 0.781172071
## 13   F4DC - FPI -3.13147289 0.001739318 0.001739318
## 14   F4DI - FPI -2.65165043 0.008009942 0.008009942
## 15    FPC - FPI -0.07576144 0.939608894 0.939608894
## 16 F1DC - M1DC -1.08591399 0.277517049 0.277517049
## 17 F1DI - M1DC  0.20203051 0.839892873 0.839892873
## 18 F4DC - M1DC -2.65165043 0.008009942 0.008009942
```

```
## 19 F4DI - M1DC -2.17182797 0.029868641 0.029868641
## 20  FPC - M1DC  0.40406102 0.686167850 0.686167850
## 21  FPI - M1DC  0.47982246 0.631353642 0.631353642
## 22 F1DC - M1DI -0.98489873 0.324673817 0.324673817
## 23 F1DI - M1DI  0.30304576 0.761854986 0.761854986
## 24 F4DC - M1DI -2.55063517 0.010752682 0.010752682
## 25 F4DI - M1DI -2.07081272 0.038376301 0.038376301
## 26  FPC - M1DI  0.50507627 0.613505266 0.613505266
## 27  FPI - M1DI  0.58083771 0.561349835 0.561349835
## 28 M1DC - M1DI  0.10101525 0.919538351 0.919538351
## 29 F1DC - M4DC  0.78286822 0.433704500 0.433704500
## 30 F1DI - M4DC  2.07081272 0.038376301 0.038376301
## 31 F4DC - M4DC -0.78286822 0.433704500 0.433704500
## 32 F4DI - M4DC -0.30304576 0.761854986 0.761854986
## 33  FPC - M4DC  2.27284323 0.023035629 0.023035629
## 34  FPI - M4DC  2.34860467 0.018843902 0.018843902
## 35 M1DC - M4DC  1.86878221 0.061653121 0.061653121
## 36 M1DI - M4DC  1.76776695 0.077099872 0.077099872
## 37 F1DC - M4DI  1.36370594 0.172660135 0.172660135
## 38 F1DI - M4DI  2.65165043 0.008009942 0.008009942
## 39 F4DC - M4DI -0.20203051 0.839892873 0.839892873
## 40 F4DI - M4DI  0.27779195 0.781172071 0.781172071
## 41  FPC - M4DI  2.85368094 0.004321591 0.004321591
## 42  FPI - M4DI  2.92944238 0.003395708 0.003395708
## 43 M1DC - M4DI  2.44961992 0.014300708 0.014300708
## 44 M1DI - M4DI  2.34860467 0.018843902 0.018843902
## 45 M4DC - M4DI  0.58083771 0.561349835 0.561349835
## 46  F1DC - MPC -1.71725933 0.085931800 0.085931800
## 47  F1DI - MPC -0.42931483 0.667694125 0.667694125
## 48  F4DC - MPC -3.28299577 0.001027102 0.001027102
## 49  F4DI - MPC -2.80317331 0.005060247 0.005060247
## 50   FPC - MPC -0.22728432 0.820202663 0.820202663
## 51   FPI - MPC -0.15152288 0.879563262 0.879563262
## 52  M1DC - MPC -0.63134534 0.527814747 0.527814747
## 53  M1DI - MPC -0.73236059 0.463948504 0.463948504
## 54  M4DC - MPC -2.50012755 0.012414860 0.012414860
## 55  M4DI - MPC -3.08096526 0.002063307 0.002063307
## 56  F1DC - MPI -1.51522882 0.129714468 0.129714468
## 57  F1DI - MPI -0.22728432 0.820202663 0.820202663
## 58  F4DC - MPI -3.08096526 0.002063307 0.002063307
## 59  F4DI - MPI -2.60114280 0.009291377 0.009291377
## 60   FPC - MPI -0.02525381 0.979852514 0.979852514
## 61   FPI - MPI  0.05050763 0.959717872 0.959717872
## 62  M1DC - MPI -0.42931483 0.667694125 0.667694125
## 63  M1DI - MPI -0.53033009 0.595883091 0.595883091
## 64  M4DC - MPI -2.29809704 0.021556267 0.021556267
## 65  M4DI - MPI -2.87893475 0.003990209 0.003990209
## 66   MPC - MPI  0.20203051 0.839892873 0.839892873
```

To prepare Figure 1

```
fig1a<-ggplot(alph, aes(x=Treatment1,y=Richness, fill=Treatment1)) +
  geom_boxplot() + geom_jitter(width=0.1,alpha=0.2) +
  ylim(20,60)
fig1a
```

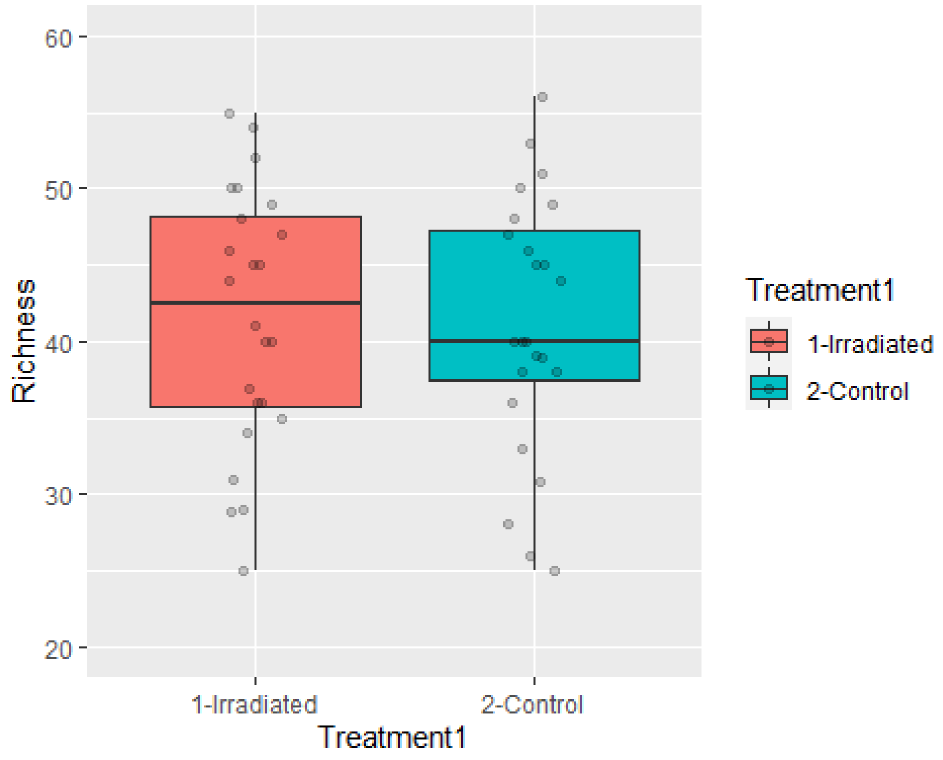


```
tiff("fig1a.tiff", width = 4, height = 4, units = 'in', res = 300)
plot(fig1a+theme_tufte() + theme(axis.line = element_line(size = 1, colour =
"black"))+ theme(legend.position = 'none') + xlab(expression(bold("Treatment"
)))+ ylab(expression(bold("Richness_index"))))
dev.off()

## png
##   2

kruskal.test(Richness~Treatment, data = alph)

##
##  Kruskal-Wallis rank sum test
##
## data:  Richness by Treatment
## Kruskal-Wallis chi-squared = 0.030724, df = 1, p-value = 0.8609

#Fig_1b

fig1b<-ggplot(alph, aes(x=Age1,y=Richness, fill=Age1)) +
  geom_boxplot() + geom_jitter(width=0.1,alpha=0.2) +
```

```
  ylim(20,60)
fig1b
```

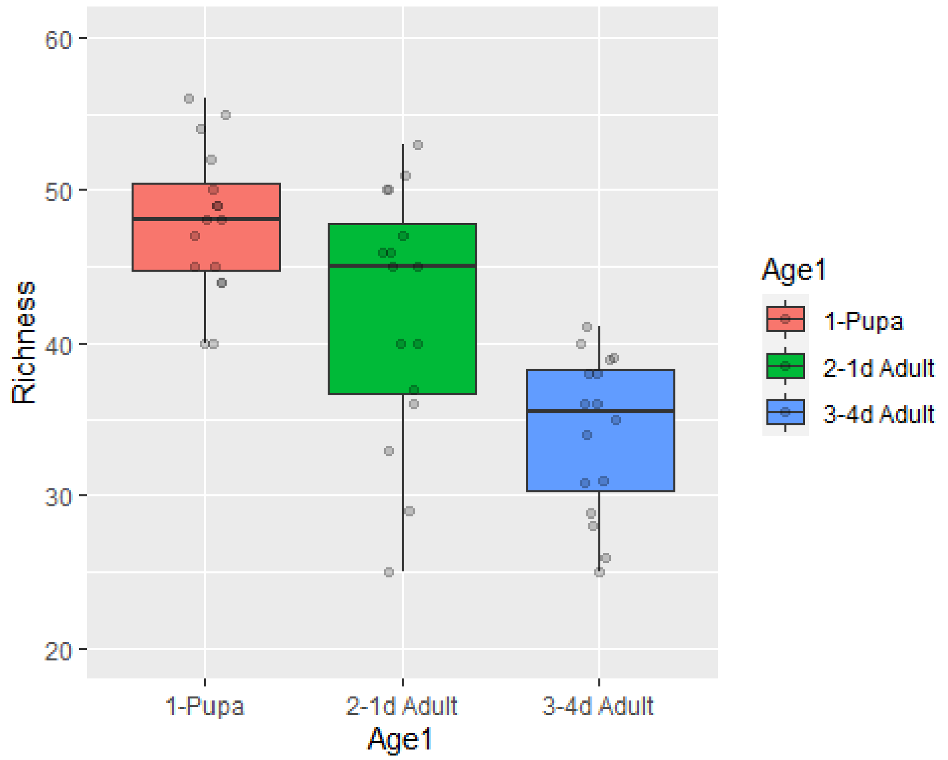


```
tiff("fig1b.tiff", width = 4, height = 4, units = 'in', res = 300)
plot(fig1b+theme_tufte() + theme(axis.line = element_line(size = 1, colour =
"black"))+ theme(legend.position = 'none') + xlab(expression(bold("Age")))+ y
lab(expression(bold("Richness_index"))))
dev.off()

## png
##   2

kruskal.test(Richness~Age, data = alph)

##
##  Kruskal-Wallis rank sum test
##
## data:  Richness by Age
## Kruskal-Wallis chi-squared = 23.451, df = 2, p-value = 8.085e-06

tmp <- dunnTest(Richness~Age, data = alph, method ="none")

## Warning: Age was coerced to a factor.

tmp

## Dunn (1964) Kruskal-Wallis multiple comparison

##   with no adjustment for p-values.
```

```
##   Comparison        Z      P.unadj        P.adj
## 1      1 - 2 1.982586 4.741372e-02 4.741372e-02
## 2      1 - 3 4.817557 1.453264e-06 1.453264e-06
## 3      2 - 3 2.834971 4.582979e-03 4.582979e-03
```

```
#Fig_1c

fig1c<-ggplot(alph, aes(x=Sex1,y=Richness, fill=Sex1)) +
  geom_boxplot() + geom_jitter(width=0.1,alpha=0.2) +
  ylim(20,60)
fig1c
```

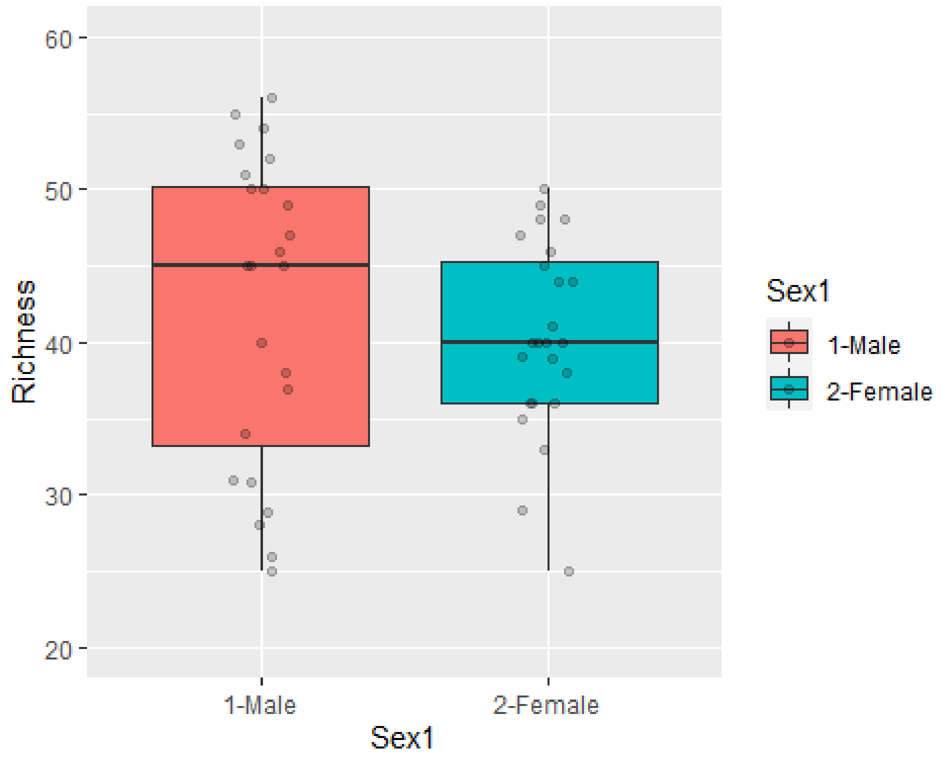


```
tiff("fig1c.tiff", width = 4, height = 4, units = 'in', res = 300)
plot(fig1c+theme_tufte() + theme(axis.line = element_line(size = 1, colour =
"black"))+ theme(legend.position = 'none') + xlab(expression(bold("Sex")))+ y
lab(expression(bold("Richness_index"))))
dev.off()

## png
##   2

kruskal.test(Richness~Sex, data = alph)

##
##  Kruskal-Wallis rank sum test
##
## data:  Richness by Sex
## Kruskal-Wallis chi-squared = 0.93935, df = 1, p-value = 0.3324
```

```r
#Fig_1d

fig1d<-ggplot(alph, aes(x=Treatment1,y=Shannon, fill=Treatment1)) +
  geom_boxplot() + geom_jitter(width=0.1,alpha=0.2) +
  ylim(1,3.5)
fig1d
```

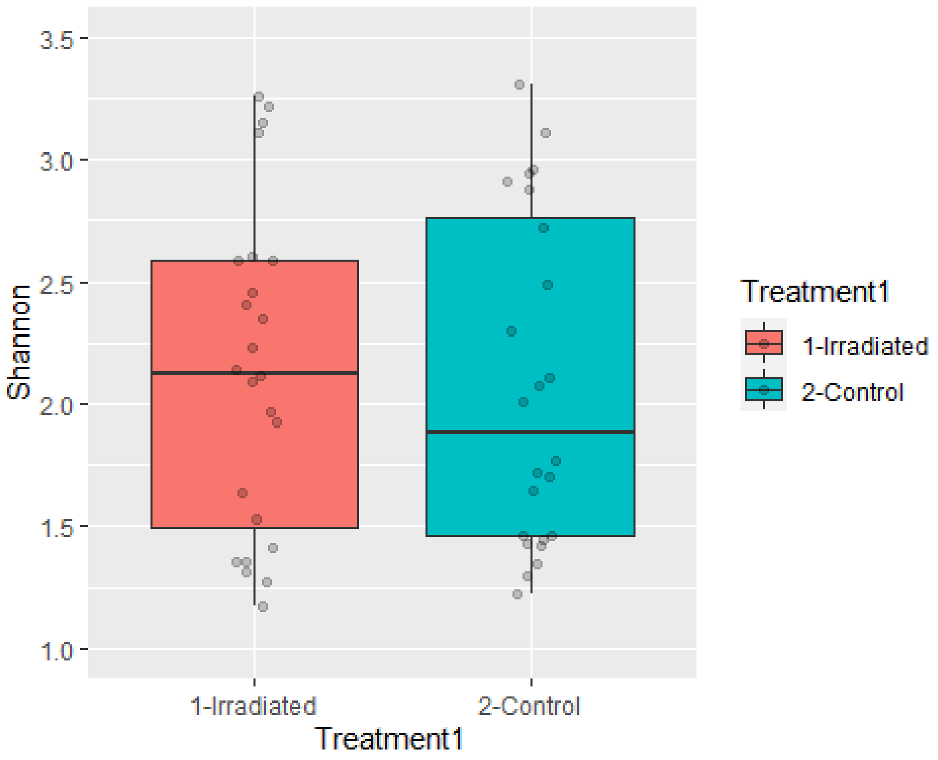


```r
tiff("fig1d.tiff", width = 4, height = 4, units = 'in', res = 300)
plot(fig1d+theme_tufte() + theme(axis.line = element_line(size = 1, colour =
"black"))+ theme(legend.position = 'none') + xlab(expression(bold("Sex")))+ y
lab(expression(bold("Shannon_index (Diversity)"))))
dev.off()

## png
##   2

with(alph,boxplot(Shannon~Treatment))
```

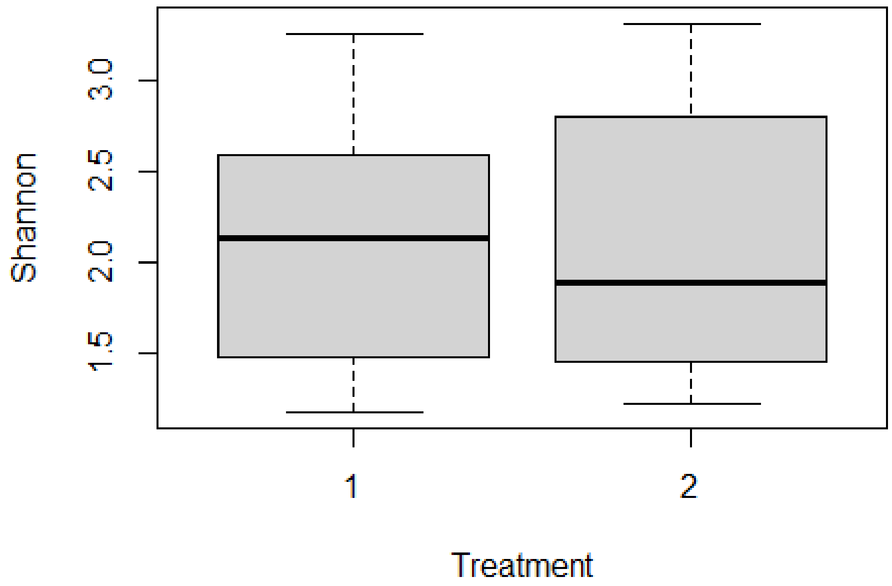


```
#Fig_1e
fig1e<-ggplot(alph, aes(x=Age1,y=Shannon, fill=Age1)) +
  geom_boxplot() + geom_jitter(width=0.1,alpha=0.2) +
  ylim(1,3.5)
fig1e
```

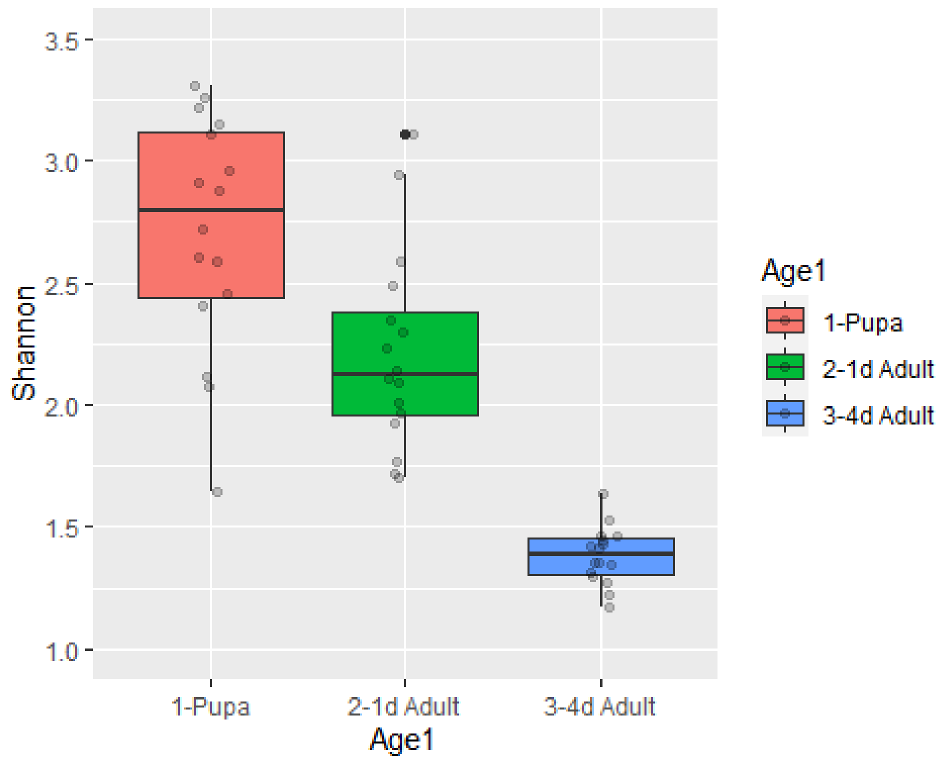


```
tiff("fig1e.tiff", width = 4, height = 4, units = 'in', res = 300)
plot(fig1e+theme_tufte() + theme(axis.line = element_line(size = 1, colour =
"black"))+ theme(legend.position = 'none') + xlab(expression(bold("Age")))+ y
lab(expression(bold("Shannon_index (Diversity)"))))
dev.off()

## png
##   2

kruskal.test(Shannon~Age, data = alph)

##
##  Kruskal-Wallis rank sum test
##
## data:  Shannon by Age
## Kruskal-Wallis chi-squared = 34.839, df = 2, p-value = 2.721e-08

tmp <- dunnTest(Shannon~Age, data = alph, method ="none")

## Warning: Age was coerced to a factor.

tmp

## Dunn (1964) Kruskal-Wallis multiple comparison
##   with no adjustment for p-values.

##   Comparison        Z      P.unadj        P.adj
## 1      1 - 2 1.868782 6.165312e-02 6.165312e-02
```

```
## 2      1 - 3 5.783123 7.332627e-09 7.332627e-09
## 3      2 - 3 3.914341 9.065141e-05 9.065141e-05
```

```
#Fig_1f
fig1f<-ggplot(alph, aes(x=Sex1,y=Shannon, fill=Sex1)) +
  geom_boxplot() + geom_jitter(width=0.1,alpha=0.2) +
  ylim(1,3.5)
fig1f
```

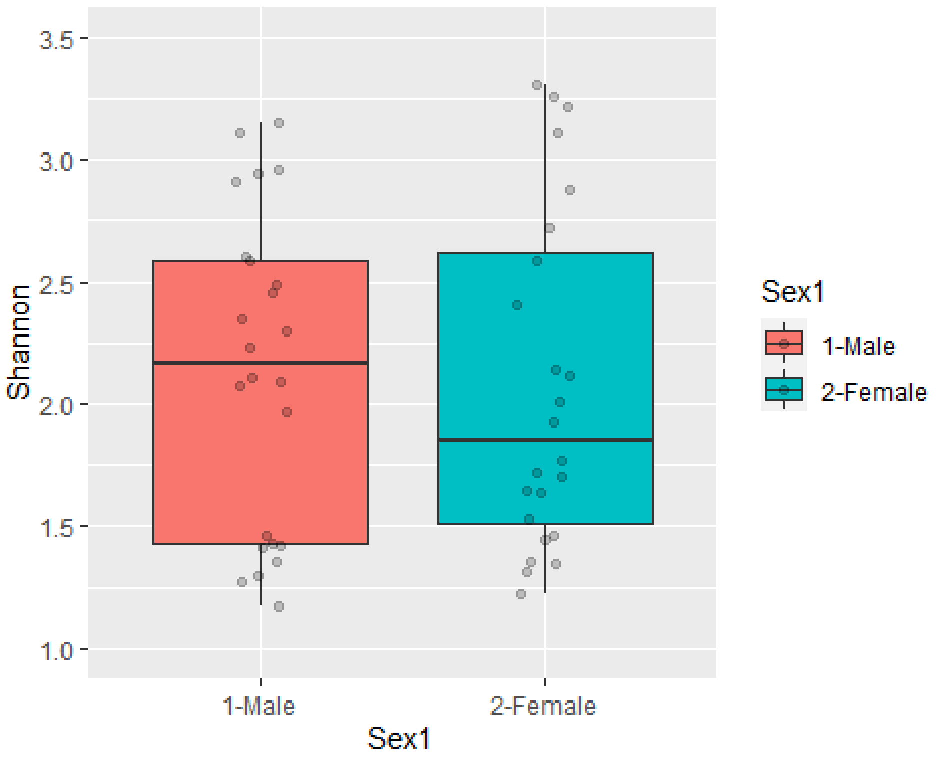


```
tiff("fig1f.tiff", width = 4, height = 4, units = 'in', res = 300)
plot(fig1f+theme_tufte() + theme(axis.line = element_line(size = 1, colour =
"black"))+ theme(legend.position = 'none') + xlab(expression(bold("Sex")))+ y
lab(expression(bold("Shannon_index (Diversity)"))))
dev.off()
```

```
## png
##   2
```

```
kruskal.test(Shannon~Sex, data = alph)
```

```
##
##  Kruskal-Wallis rank sum test
##
## data:  Shannon by Sex
## Kruskal-Wallis chi-squared = 0.042517, df = 1, p-value = 0.8366
```

To prepare supplemetary table 3

Compare the indixes between the two regions for all samples

```
setwd("C:/Users/abdallaa/OneDrive - IAEA/My/Kostas_paper 2020/Final_version_F
eb_2021/Revission/raw_data")
da1 <- read.csv("table_S3.csv")
#fig1$Time=as.factor(fig1$Time)
da1=na.omit(da1)
head(da1)
```

```
##   X         BarcodeName Pielou.s.evenness Richness  Shannon   Simpson Regio
ns
## 1 1 1_Aa_fe_1d_con_1_1         0.4493868 17.00000 1.273209 0.6236611
R1
## 2 2 2_Aa_fe_1d_con_2_1         0.3981715 29.00000 1.340761 0.6258106
R1
## 3 3 3_Aa_fe_1d_con_3_1         0.5239999 38.00000 1.906095 0.7295495
R1
## 4 4 4_Aa_fe_1d_con_4_1         0.4054128 38.00000 1.474724 0.6385121
R1
## 5 5 5_Aa_fe_1d_ira_1_1         0.4144742 23.99715 1.317221 0.6772537
R1
## 6 6 6_Aa_fe_1d_ira_2_1         0.6085897 49.00000 2.368522 0.8050048
R1
##      Sex Age Stage  Treatment
## 1 Female  1D Adult    Control
## 2 Female  1D Adult    Control
## 3 Female  1D Adult    Control
## 4 Female  1D Adult    Control
## 5 Female  1D Adult Irradiated
## 6 Female  1D Adult Irradiated
```

```
kruskal.test(Pielou.s.evenness~Regions, data=da1)
```

```
##
##  Kruskal-Wallis rank sum test
##
## data:  Pielou.s.evenness by Regions
## Kruskal-Wallis chi-squared = 0.24828, df = 1, p-value = 0.6183
```

```
kruskal.test(Richness~Regions, data=da1)
```

```
##
##  Kruskal-Wallis rank sum test
##
## data:  Richness by Regions
## Kruskal-Wallis chi-squared = 67.223, df = 1, p-value = 2.425e-16
```

```
kruskal.test(Shannon~Regions, data=da1)
```

```
##
##  Kruskal-Wallis rank sum test
##
## data:  Shannon by Regions
## Kruskal-Wallis chi-squared = 8.3776, df = 1, p-value = 0.003799
```

```
kruskal.test(Simpson~Regions, data=da1)
```

```
##
##  Kruskal-Wallis rank sum test
##
## data:  Simpson by Regions
## Kruskal-Wallis chi-squared = 3.6019, df = 1, p-value = 0.05772
```

Compare the indicies between the two regions for each samples

```
da2 <- subset(da1, Sex=="Male" & Age=="Pupa" & Treatment=="Irradiated")
head(da2)
```

```
##     X       BarcodeName Pielou.s.evenness Richness  Shannon   Simpson Regi
ons
## 45 45 45_Aa_m_p_ira_1_1         0.5590665       50 2.187081 0.7105980
R1
## 46 46 46_Aa_m_p_ira_2_1         0.7803966       49 3.037163 0.9230362
R1
## 47 47 47_Aa_m_p_ira_3_1         0.4986671       48 1.930440 0.7439916
R1
## 48 48 48_Aa_m_p_ira_4_1         0.6295169       43 2.367739 0.7820541
R1
## 93 45 45_Aa_m_p_ira_1_1         0.6668527       20 1.997712 0.7737144
R2
## 94 46 46_Aa_m_p_ira_2_1         0.7223271       23 2.264852 0.8450655
R2
##     Sex  Age Stage  Treatment
## 45 Male Pupa  Pupa Irradiated
## 46 Male Pupa  Pupa Irradiated
## 47 Male Pupa  Pupa Irradiated
## 48 Male Pupa  Pupa Irradiated
## 93 Male Pupa  Pupa Irradiated
## 94 Male Pupa  Pupa Irradiated
```

```
# kruscal.tset
kruskal.test(Pielou.s.evenness~Regions, data=da2)
```

```
##
##  Kruskal-Wallis rank sum test
##
## data:  Pielou.s.evenness by Regions
## Kruskal-Wallis chi-squared = 0.083333, df = 1, p-value = 0.7728
```

```
kruskal.test(Richness~Regions, data=da2)
```

```
##
##  Kruskal-Wallis rank sum test
##
## data:  Richness by Regions
## Kruskal-Wallis chi-squared = 5.3333, df = 1, p-value = 0.02092
```

```
kruskal.test(Shannon~Regions, data=da2)
```

```
##
##  Kruskal-Wallis rank sum test
##
## data:  Shannon by Regions
## Kruskal-Wallis chi-squared = 2.0833, df = 1, p-value = 0.1489
```

```
kruskal.test(Simpson~Regions, data=da2)
```

```
##
##  Kruskal-Wallis rank sum test
##
## data:  Simpson by Regions
## Kruskal-Wallis chi-squared = 0.33333, df = 1, p-value = 0.5637
```

```
da3 <- subset(da1, Sex=="Male" & Age=="Pupa" & Treatment=="Control")
head(da2)
```

```
##     X       BarcodeName Pielou.s.evenness Richness  Shannon   Simpson Regi
ons
## 45 45 45_Aa_m_p_ira_1_1         0.5590665       50 2.187081 0.7105980
R1
## 46 46 46_Aa_m_p_ira_2_1         0.7803966       49 3.037163 0.9230362
R1
## 47 47 47_Aa_m_p_ira_3_1         0.4986671       48 1.930440 0.7439916
R1
## 48 48 48_Aa_m_p_ira_4_1         0.6295169       43 2.367739 0.7820541
R1
## 93 45 45_Aa_m_p_ira_1_1         0.6668527       20 1.997712 0.7737144
R2
## 94 46 46_Aa_m_p_ira_2_1         0.7223271       23 2.264852 0.8450655
R2
##     Sex  Age Stage  Treatment
## 45 Male Pupa  Pupa Irradiated
## 46 Male Pupa  Pupa Irradiated
## 47 Male Pupa  Pupa Irradiated
## 48 Male Pupa  Pupa Irradiated
## 93 Male Pupa  Pupa Irradiated
## 94 Male Pupa  Pupa Irradiated
```

```
# kruscal.tset
kruskal.test(Pielou.s.evenness~Regions, data=da3)
```

```
##
##  Kruskal-Wallis rank sum test
```

```
##
## data:  Pielou.s.evenness by Regions
## Kruskal-Wallis chi-squared = 0.098182, df = 1, p-value = 0.754
```

```
kruskal.test(Richness~Regions, data=da3)
```

```
##
##  Kruskal-Wallis rank sum test
##
## data:  Richness by Regions
## Kruskal-Wallis chi-squared = 6.8598, df = 1, p-value = 0.008816
```

```
kruskal.test(Shannon~Regions, data=da3)
```

```
##
##  Kruskal-Wallis rank sum test
##
## data:  Shannon by Regions
## Kruskal-Wallis chi-squared = 3.9382, df = 1, p-value = 0.0472
```

```
kruskal.test(Simpson~Regions, data=da3)
```

```
##
##  Kruskal-Wallis rank sum test
##
## data:  Simpson by Regions
## Kruskal-Wallis chi-squared = 1.32, df = 1, p-value = 0.2506
```

```
#==============================================================
da4 <- subset(da1, Sex=="Female" & Age=="Pupa" & Treatment=="Irradiated")
head(da2)
```

```
##     X       BarcodeName Pielou.s.evenness Richness  Shannon   Simpson Regi
ons
## 45 45 45_Aa_m_p_ira_1_1         0.5590665       50 2.187081 0.7105980
R1
## 46 46 46_Aa_m_p_ira_2_1         0.7803966       49 3.037163 0.9230362
R1
## 47 47 47_Aa_m_p_ira_3_1         0.4986671       48 1.930440 0.7439916
R1
## 48 48 48_Aa_m_p_ira_4_1         0.6295169       43 2.367739 0.7820541
R1
## 93 45 45_Aa_m_p_ira_1_1         0.6668527       20 1.997712 0.7737144
R2
## 94 46 46_Aa_m_p_ira_2_1         0.7223271       23 2.264852 0.8450655
R2
##     Sex  Age Stage  Treatment
## 45 Male Pupa  Pupa Irradiated
## 46 Male Pupa  Pupa Irradiated
## 47 Male Pupa  Pupa Irradiated
## 48 Male Pupa  Pupa Irradiated
```

```
## 93 Male Pupa  Pupa Irradiated
## 94 Male Pupa  Pupa Irradiated

# kruscal.tset
kruskal.test(Pielou.s.evenness~Regions, data=da4)

##
##  Kruskal-Wallis rank sum test
##
## data:  Pielou.s.evenness by Regions
## Kruskal-Wallis chi-squared = 0, df = 1, p-value = 1

kruskal.test(Richness~Regions, data=da4)

##
##  Kruskal-Wallis rank sum test
##
## data:  Richness by Regions
## Kruskal-Wallis chi-squared = 5.3333, df = 1, p-value = 0.02092

kruskal.test(Shannon~Regions, data=da4)

##
##  Kruskal-Wallis rank sum test
##
## data:  Shannon by Regions
## Kruskal-Wallis chi-squared = 1.3333, df = 1, p-value = 0.2482

kruskal.test(Simpson~Regions, data=da4)

##
##  Kruskal-Wallis rank sum test
##
## data:  Simpson by Regions
## Kruskal-Wallis chi-squared = 0, df = 1, p-value = 1

da5 <- subset(da1, Sex=="Female" & Age=="Pupa" & Treatment=="Control")
head(da2)

##     X       BarcodeName Pielou.s.evenness Richness  Shannon   Simpson Regi
ons
## 45 45 45_Aa_m_p_ira_1_1         0.5590665       50 2.187081 0.7105980
R1
## 46 46 46_Aa_m_p_ira_2_1         0.7803966       49 3.037163 0.9230362
R1
## 47 47 47_Aa_m_p_ira_3_1         0.4986671       48 1.930440 0.7439916
R1
## 48 48 48_Aa_m_p_ira_4_1         0.6295169       43 2.367739 0.7820541
R1
## 93 45 45_Aa_m_p_ira_1_1         0.6668527       20 1.997712 0.7737144
R2
## 94 46 46_Aa_m_p_ira_2_1         0.7223271       23 2.264852 0.8450655
```

```
R2
##     Sex  Age Stage  Treatment
## 45 Male Pupa  Pupa Irradiated
## 46 Male Pupa  Pupa Irradiated
## 47 Male Pupa  Pupa Irradiated
## 48 Male Pupa  Pupa Irradiated
## 93 Male Pupa  Pupa Irradiated
## 94 Male Pupa  Pupa Irradiated
```

```
# kruscal.tset
kruskal.test(Pielou.s.evenness~Regions, data=da5)
```

```
##
##  Kruskal-Wallis rank sum test
##
## data:  Pielou.s.evenness by Regions
## Kruskal-Wallis chi-squared = 0.33333, df = 1, p-value = 0.5637
```

```
kruskal.test(Richness~Regions, data=da5)
```

```
##
##  Kruskal-Wallis rank sum test
##
## data:  Richness by Regions
## Kruskal-Wallis chi-squared = 5.6, df = 1, p-value = 0.01796
```

```
kruskal.test(Shannon~Regions, data=da5)
```

```
##
##  Kruskal-Wallis rank sum test
##
## data:  Shannon by Regions
## Kruskal-Wallis chi-squared = 4.0833, df = 1, p-value = 0.04331
```

```
kruskal.test(Simpson~Regions, data=da5)
```

```
##
##  Kruskal-Wallis rank sum test
##
## data:  Simpson by Regions
## Kruskal-Wallis chi-squared = 1.3333, df = 1, p-value = 0.2482
```

```
#====================================================================

da6 <- subset(da1, Sex=="Male" & Age=="1D" & Treatment=="Irradiated")
head(da2)
```

```
##     X       BarcodeName Pielou.s.evenness Richness  Shannon   Simpson Regi
ons
## 45 45 45_Aa_m_p_ira_1_1         0.5590665       50 2.187081 0.7105980
R1
## 46 46 46_Aa_m_p_ira_2_1         0.7803966       49 3.037163 0.9230362
```

```
R1
## 47 47 47_Aa_m_p_ira_3_1         0.4986671       48 1.930440 0.7439916
R1
## 48 48 48_Aa_m_p_ira_4_1         0.6295169       43 2.367739 0.7820541
R1
## 93 45 45_Aa_m_p_ira_1_1         0.6668527       20 1.997712 0.7737144
R2
## 94 46 46_Aa_m_p_ira_2_1         0.7223271       23 2.264852 0.8450655
R2
##     Sex  Age Stage  Treatment
## 45 Male Pupa  Pupa Irradiated
## 46 Male Pupa  Pupa Irradiated
## 47 Male Pupa  Pupa Irradiated
## 48 Male Pupa  Pupa Irradiated
## 93 Male Pupa  Pupa Irradiated
## 94 Male Pupa  Pupa Irradiated

# kruscal.tset
kruskal.test(Pielou.s.evenness~Regions, data=da6)

##
##  Kruskal-Wallis rank sum test
##
## data:  Pielou.s.evenness by Regions
## Kruskal-Wallis chi-squared = 0.33333, df = 1, p-value = 0.5637

kruskal.test(Richness~Regions, data=da6)

##
##  Kruskal-Wallis rank sum test
##
## data:  Richness by Regions
## Kruskal-Wallis chi-squared = 5.3333, df = 1, p-value = 0.02092

kruskal.test(Shannon~Regions, data=da6)

##
##  Kruskal-Wallis rank sum test
##
## data:  Shannon by Regions
## Kruskal-Wallis chi-squared = 3, df = 1, p-value = 0.08326

kruskal.test(Simpson~Regions, data=da6)

##
##  Kruskal-Wallis rank sum test
##
## data:  Simpson by Regions
## Kruskal-Wallis chi-squared = 2.0833, df = 1, p-value = 0.1489

da7 <- subset(da1, Sex=="Male" & Age=="1D" & Treatment=="Control")
head(da2)
```

```
##     X       BarcodeName Pielou.s.evenness Richness  Shannon   Simpson Regi
ons
## 45 45 45_Aa_m_p_ira_1_1         0.5590665       50 2.187081 0.7105980
R1
## 46 46 46_Aa_m_p_ira_2_1         0.7803966       49 3.037163 0.9230362
R1
## 47 47 47_Aa_m_p_ira_3_1         0.4986671       48 1.930440 0.7439916
R1
## 48 48 48_Aa_m_p_ira_4_1         0.6295169       43 2.367739 0.7820541
R1
## 93 45 45_Aa_m_p_ira_1_1         0.6668527       20 1.997712 0.7737144
R2
## 94 46 46_Aa_m_p_ira_2_1         0.7223271       23 2.264852 0.8450655
R2
##     Sex  Age Stage  Treatment
## 45 Male Pupa  Pupa Irradiated
## 46 Male Pupa  Pupa Irradiated
## 47 Male Pupa  Pupa Irradiated
## 48 Male Pupa  Pupa Irradiated
## 93 Male Pupa  Pupa Irradiated
## 94 Male Pupa  Pupa Irradiated
```

```
# kruscal.tset
kruskal.test(Pielou.s.evenness~Regions, data=da7)
```

```
##
##  Kruskal-Wallis rank sum test
##
## data:  Pielou.s.evenness by Regions
## Kruskal-Wallis chi-squared = 0.047619, df = 1, p-value = 0.8273
```

```
kruskal.test(Richness~Regions, data=da7)
```

```
##
##  Kruskal-Wallis rank sum test
##
## data:  Richness by Regions
## Kruskal-Wallis chi-squared = 3.8571, df = 1, p-value = 0.04953
```

```
kruskal.test(Shannon~Regions, data=da7)
```

```
##
##  Kruskal-Wallis rank sum test
##
## data:  Shannon by Regions
## Kruskal-Wallis chi-squared = 0.047619, df = 1, p-value = 0.8273
```

```
kruskal.test(Simpson~Regions, data=da7)
```

```
##
##  Kruskal-Wallis rank sum test
##
```

```
## data:  Simpson by Regions
## Kruskal-Wallis chi-squared = 0.047619, df = 1, p-value = 0.8273

#==============================================================
da8 <- subset(da1, Sex=="Female" & Age=="1D" & Treatment=="Irradiated")
head(da2)

##     X       BarcodeName Pielou.s.evenness Richness  Shannon   Simpson Regi
ons
## 45 45 45_Aa_m_p_ira_1_1         0.5590665       50 2.187081 0.7105980
R1
## 46 46 46_Aa_m_p_ira_2_1         0.7803966       49 3.037163 0.9230362
R1
## 47 47 47_Aa_m_p_ira_3_1         0.4986671       48 1.930440 0.7439916
R1
## 48 48 48_Aa_m_p_ira_4_1         0.6295169       43 2.367739 0.7820541
R1
## 93 45 45_Aa_m_p_ira_1_1         0.6668527       20 1.997712 0.7737144
R2
## 94 46 46_Aa_m_p_ira_2_1         0.7223271       23 2.264852 0.8450655
R2
##     Sex  Age Stage  Treatment
## 45 Male Pupa  Pupa Irradiated
## 46 Male Pupa  Pupa Irradiated
## 47 Male Pupa  Pupa Irradiated
## 48 Male Pupa  Pupa Irradiated
## 93 Male Pupa  Pupa Irradiated
## 94 Male Pupa  Pupa Irradiated

# kruscal.tset
kruskal.test(Pielou.s.evenness~Regions, data=da8)

## 
##  Kruskal-Wallis rank sum test
## 
## data:  Pielou.s.evenness by Regions
## Kruskal-Wallis chi-squared = 0.33333, df = 1, p-value = 0.5637

kruskal.test(Richness~Regions, data=da8)

## 
##  Kruskal-Wallis rank sum test
## 
## data:  Richness by Regions
## Kruskal-Wallis chi-squared = 5.3333, df = 1, p-value = 0.02092

kruskal.test(Shannon~Regions, data=da8)

## 
##  Kruskal-Wallis rank sum test
## 
```

```
## data:  Shannon by Regions
## Kruskal-Wallis chi-squared = 2.0833, df = 1, p-value = 0.1489
```

```
kruskal.test(Simpson~Regions, data=da8)
```

```
##
##  Kruskal-Wallis rank sum test
##
## data:  Simpson by Regions
## Kruskal-Wallis chi-squared = 2.0833, df = 1, p-value = 0.1489
```

```
da9 <- subset(da1, Sex=="Female" & Age=="1D" & Treatment=="Control")
head(da2)
```

```
##     X       BarcodeName Pielou.s.evenness Richness  Shannon   Simpson Regi
ons
## 45 45 45_Aa_m_p_ira_1_1         0.5590665       50 2.187081 0.7105980
R1
## 46 46 46_Aa_m_p_ira_2_1         0.7803966       49 3.037163 0.9230362
R1
## 47 47 47_Aa_m_p_ira_3_1         0.4986671       48 1.930440 0.7439916
R1
## 48 48 48_Aa_m_p_ira_4_1         0.6295169       43 2.367739 0.7820541
R1
## 93 45 45_Aa_m_p_ira_1_1         0.6668527       20 1.997712 0.7737144
R2
## 94 46 46_Aa_m_p_ira_2_1         0.7223271       23 2.264852 0.8450655
R2
##     Sex  Age Stage  Treatment
## 45 Male Pupa  Pupa Irradiated
## 46 Male Pupa  Pupa Irradiated
## 47 Male Pupa  Pupa Irradiated
## 48 Male Pupa  Pupa Irradiated
## 93 Male Pupa  Pupa Irradiated
## 94 Male Pupa  Pupa Irradiated
```

```
# kruscal.tset
kruskal.test(Pielou.s.evenness~Regions, data=da9)
```

```
##
##  Kruskal-Wallis rank sum test
##
## data:  Pielou.s.evenness by Regions
## Kruskal-Wallis chi-squared = 2.0833, df = 1, p-value = 0.1489
```

```
kruskal.test(Richness~Regions, data=da9)
```

```
##
##  Kruskal-Wallis rank sum test
##
## data:  Richness by Regions
## Kruskal-Wallis chi-squared = 5.3333, df = 1, p-value = 0.02092
```

```
kruskal.test(Shannon~Regions, data=da9)

##
##  Kruskal-Wallis rank sum test
##
## data:  Shannon by Regions
## Kruskal-Wallis chi-squared = 5.3333, df = 1, p-value = 0.02092

kruskal.test(Simpson~Regions, data=da9)

##
##  Kruskal-Wallis rank sum test
##
## data:  Simpson by Regions
## Kruskal-Wallis chi-squared = 3, df = 1, p-value = 0.08326

#=====================================================================

da10 <- subset(da1, Sex=="Male" & Age=="4D" & Treatment=="Irradiated")
head(da2)

##     X       BarcodeName Pielou.s.evenness Richness  Shannon   Simpson Regi
ons
## 45 45 45_Aa_m_p_ira_1_1         0.5590665       50 2.187081 0.7105980
R1
## 46 46 46_Aa_m_p_ira_2_1         0.7803966       49 3.037163 0.9230362
R1
## 47 47 47_Aa_m_p_ira_3_1         0.4986671       48 1.930440 0.7439916
R1
## 48 48 48_Aa_m_p_ira_4_1         0.6295169       43 2.367739 0.7820541
R1
## 93 45 45_Aa_m_p_ira_1_1         0.6668527       20 1.997712 0.7737144
R2
## 94 46 46_Aa_m_p_ira_2_1         0.7223271       23 2.264852 0.8450655
R2
##     Sex  Age Stage  Treatment
## 45 Male Pupa  Pupa Irradiated
## 46 Male Pupa  Pupa Irradiated
## 47 Male Pupa  Pupa Irradiated
## 48 Male Pupa  Pupa Irradiated
## 93 Male Pupa  Pupa Irradiated
## 94 Male Pupa  Pupa Irradiated

# kruscal.tset
kruskal.test(Pielou.s.evenness~Regions, data=da10)

##
##  Kruskal-Wallis rank sum test
##
## data:  Pielou.s.evenness by Regions
## Kruskal-Wallis chi-squared = 3, df = 1, p-value = 0.08326
```

```
kruskal.test(Richness~Regions, data=da10)

##
##  Kruskal-Wallis rank sum test
##
## data:  Richness by Regions
## Kruskal-Wallis chi-squared = 5.3976, df = 1, p-value = 0.02016

kruskal.test(Shannon~Regions, data=da10)

##
##  Kruskal-Wallis rank sum test
##
## data:  Shannon by Regions
## Kruskal-Wallis chi-squared = 0.083333, df = 1, p-value = 0.7728

kruskal.test(Simpson~Regions, data=da10)

##
##  Kruskal-Wallis rank sum test
##
## data:  Simpson by Regions
## Kruskal-Wallis chi-squared = 1.3333, df = 1, p-value = 0.2482

da11 <- subset(da1, Sex=="Male" & Age=="4D" & Treatment=="Control")
head(da2)

##     X       BarcodeName Pielou.s.evenness Richness  Shannon   Simpson Regi
ons
## 45 45 45_Aa_m_p_ira_1_1         0.5590665       50 2.187081 0.7105980
R1
## 46 46 46_Aa_m_p_ira_2_1         0.7803966       49 3.037163 0.9230362
R1
## 47 47 47_Aa_m_p_ira_3_1         0.4986671       48 1.930440 0.7439916
R1
## 48 48 48_Aa_m_p_ira_4_1         0.6295169       43 2.367739 0.7820541
R1
## 93 45 45_Aa_m_p_ira_1_1         0.6668527       20 1.997712 0.7737144
R2
## 94 46 46_Aa_m_p_ira_2_1         0.7223271       23 2.264852 0.8450655
R2
##     Sex  Age Stage  Treatment
## 45 Male Pupa  Pupa Irradiated
## 46 Male Pupa  Pupa Irradiated
## 47 Male Pupa  Pupa Irradiated
## 48 Male Pupa  Pupa Irradiated
## 93 Male Pupa  Pupa Irradiated
## 94 Male Pupa  Pupa Irradiated

# kruscal.tset
kruskal.test(Pielou.s.evenness~Regions, data=da11)
```

```
##
##  Kruskal-Wallis rank sum test
##
## data:  Pielou.s.evenness by Regions
## Kruskal-Wallis chi-squared = 0, df = 1, p-value = 1

kruskal.test(Richness~Regions, data=da11)

##
##  Kruskal-Wallis rank sum test
##
## data:  Richness by Regions
## Kruskal-Wallis chi-squared = 5.3333, df = 1, p-value = 0.02092

kruskal.test(Shannon~Regions, data=da11)

##
##  Kruskal-Wallis rank sum test
##
## data:  Shannon by Regions
## Kruskal-Wallis chi-squared = 2.0833, df = 1, p-value = 0.1489

kruskal.test(Simpson~Regions, data=da11)

##
##  Kruskal-Wallis rank sum test
##
## data:  Simpson by Regions
## Kruskal-Wallis chi-squared = 0.33333, df = 1, p-value = 0.5637

#=============================================================
da12 <- subset(da1, Sex=="Female" & Age=="4D" & Treatment=="Irradiated")
head(da2)

##      X        BarcodeName Pielou.s.evenness Richness  Shannon   Simpson Regi
ons
## 45 45 45_Aa_m_p_ira_1_1         0.5590665       50 2.187081 0.7105980
R1
## 46 46 46_Aa_m_p_ira_2_1         0.7803966       49 3.037163 0.9230362
R1
## 47 47 47_Aa_m_p_ira_3_1         0.4986671       48 1.930440 0.7439916
R1
## 48 48 48_Aa_m_p_ira_4_1         0.6295169       43 2.367739 0.7820541
R1
## 93 45 45_Aa_m_p_ira_1_1         0.6668527       20 1.997712 0.7737144
R2
## 94 46 46_Aa_m_p_ira_2_1         0.7223271       23 2.264852 0.8450655
R2
##     Sex  Age Stage  Treatment
## 45 Male Pupa  Pupa Irradiated
## 46 Male Pupa  Pupa Irradiated
## 47 Male Pupa  Pupa Irradiated
```

```
## 48 Male Pupa  Pupa Irradiated
## 93 Male Pupa  Pupa Irradiated
## 94 Male Pupa  Pupa Irradiated

# kruscal.tset
kruskal.test(Pielou.s.evenness~Regions, data=da12)

##
##  Kruskal-Wallis rank sum test
##
## data:  Pielou.s.evenness by Regions
## Kruskal-Wallis chi-squared = 0.33333, df = 1, p-value = 0.5637

kruskal.test(Richness~Regions, data=da12)

##
##  Kruskal-Wallis rank sum test
##
## data:  Richness by Regions
## Kruskal-Wallis chi-squared = 5.4634, df = 1, p-value = 0.01942

kruskal.test(Shannon~Regions, data=da12)

##
##  Kruskal-Wallis rank sum test
##
## data:  Shannon by Regions
## Kruskal-Wallis chi-squared = 1.3333, df = 1, p-value = 0.2482

kruskal.test(Simpson~Regions, data=da12)

##
##  Kruskal-Wallis rank sum test
##
## data:  Simpson by Regions
## Kruskal-Wallis chi-squared = 0, df = 1, p-value = 1

da13 <- subset(da1, Sex=="Female" & Age=="4D" & Treatment=="Control")
head(da2)

##     X       BarcodeName Pielou.s.evenness Richness  Shannon   Simpson Regi
ons
## 45 45 45_Aa_m_p_ira_1_1         0.5590665       50 2.187081 0.7105980
R1
## 46 46 46_Aa_m_p_ira_2_1         0.7803966       49 3.037163 0.9230362
R1
## 47 47 47_Aa_m_p_ira_3_1         0.4986671       48 1.930440 0.7439916
R1
## 48 48 48_Aa_m_p_ira_4_1         0.6295169       43 2.367739 0.7820541
R1
## 93 45 45_Aa_m_p_ira_1_1         0.6668527       20 1.997712 0.7737144
R2
```

```
## 94 46 46_Aa_m_p_ira_2_1         0.7223271       23 2.264852 0.8450655
R2
##     Sex  Age Stage  Treatment
## 45 Male Pupa  Pupa Irradiated
## 46 Male Pupa  Pupa Irradiated
## 47 Male Pupa  Pupa Irradiated
## 48 Male Pupa  Pupa Irradiated
## 93 Male Pupa  Pupa Irradiated
## 94 Male Pupa  Pupa Irradiated

# kruscal.tset
kruskal.test(Pielou.s.evenness~Regions, data=da13)

##
##  Kruskal-Wallis rank sum test
##
## data:  Pielou.s.evenness by Regions
## Kruskal-Wallis chi-squared = 0.083333, df = 1, p-value = 0.7728

kruskal.test(Richness~Regions, data=da13)

##
##  Kruskal-Wallis rank sum test
##
## data:  Richness by Regions
## Kruskal-Wallis chi-squared = 5.3976, df = 1, p-value = 0.02016

kruskal.test(Shannon~Regions, data=da13)

##
##  Kruskal-Wallis rank sum test
##
## data:  Shannon by Regions
## Kruskal-Wallis chi-squared = 3, df = 1, p-value = 0.08326

kruskal.test(Simpson~Regions, data=da13)

##
##  Kruskal-Wallis rank sum test
##
## data:  Simpson by Regions
## Kruskal-Wallis chi-squared = 0.75, df = 1, p-value = 0.3865
```
