# Supplementary material for: The Effect of Radiation on the Gut Bacteriome of Aedes albopictus
Source: Front Microbiol. 2021 Jul 8;12:671699. doi: 10.3389/fmicb.2021.671699 (PMC8299835; doi:10.3389/fmicb.2021.671699)
Supplement: Supplementary file 2 [file Data_Sheet_1.pdf]

Supplementary files of manuscript:

The effect of radiation on the gut bacteriome of *Aedes albopictus*

Dongjing Zhang[1,2,4 #], Shi Chen[1,3 #], Adly Abd-Alla[1] and Kostas Bourtzis[1,*]

[1] Insect Pest Control Laboratory, Joint FAO/IAEA Division of Nuclear Techniques in Food and Agriculture, Vienna, Austria

[2] Key Laboratory of Tropical Disease Control of the Ministry of Education, Sun Yat-sen University–Michigan State University Joint Center of Vector Control for Tropical Diseases, Zhongshan School of Medicine, Sun Yat-sen University, Guangzhou, China

[3] Institute of Biological Control, Fujian Agricultural and Forestry University, Fuzhou, Fujian Province, China

[4] Chinese Atomic Energy Agency Center of Excellence on Nuclear Technology Applications for Insect Control, Sun Yat-sen University, Guangzhou, China

# These authors have contributed equally to this work.

*** Corresponding Author:**

Email: K.Bourtzis@iaea.org

Keywords: sterile insect technique, vector control, dengue, 16S *rRNA* gene, Aeromonas, *Elizabethkingia*

Supplementary files

Supplementary Table 1. Summary of the analyzed samples

ID	Age	Diet	Sex	Irradiation (40 Gy)
MPI	24-36 hours old pupa	Nil	Male	Yes
MPC	24-36 hours old pupa	Nil	Male	No
FPI	24-36 hours old pupa	Nil	Female	Yes
FPC	24-36 hours old pupa	Nil	Female	No
1DMI	1-day-old adult	Nil	Male	Yes
1DMC	1-day-old adult	Nil	Male	No
1DFI	1-day-old adult	Nil	Female	Yes
1DFC	1-day-old adult	Nil	Female	No
4DMI	4-day-old adult	Sugar	Male	Yes
4DMC	4-day-old adult	Sugar	Male	No
4DFI	4-day-old adult	Sugar	Female	Yes
4DFC	4-day-old adult	Sugar	Female	No

Supplementary Table 2. Primers used in the qPCR experiments

Target taxon	Primer	Sequence (5'-3')	T(A)	T(M)	Reference
Aeromonas	Aerunibes-3	ATCACCGGCAGTCT CCCTTGAG	66 ℃	86 ºC	This study
	Aerunibes-5	GGGAGTGCCTTCGG GAATCAGA			
Elizabethkingia	Elic6-3	TCAGGCTTCCACCC ATTGTCCA	66 ℃	85 ºC	This study
	Elic6-5	TAATTGAGAGCGG CGTACGGGT			
Enterococcus	Entbes-3	TTGCTAGAGTGCCC AACTGAAT	65 ℃	84 ºC	This study
	Entbes-5	CCCTTACCAGGTCT TGACATCC			
Ribosomal protein S6 gene	Rps6-qpcrF	CGTCGTCAGGAAC GTATTCG	58 ℃	86 ºC	(Zheng et al. 2019)
	Rps6-qpcrR	TCTTGGCAGCCTTG ACAGC			

Supplementary Table 3. Statistical value for the comparison between the two regions (R1 and R2) of 16S *rRNA* gene

Sample	Pielou.s.evenness	Species richness	Species diversity indices	
			Shannon	Simpson reciprocal
MPI	0.7728	0.02092*	0.1489	0.5637
MPC	0.754	0.008816*	0.0472*	0.2506
FPI	1	0.02092*	0.2482	1
FPC	0.5637	0.01796*	0.04331*	0.2482
1DMI	0.5637	0.02092*	0.08326	0.1489
1DMC	0.8273	0.04953*	0.8273	0.8273
1DFI	0.5637	0.02092*	0.1489	0.1489
1DFC	0.1489	0.02092*	0.02092*	0.08326
4DMI	0.08326	0.02016*	0.7728	0.2482
4DMC	1	0.02092*	0.1489	0.5637
4DFI	0.5637	0.01942*	0.2482	1
4DFC	0.7728	0.02016*	0.08326	0.3865
All samples	0.6183	2.425e-16*	0.003799*	0.05772

For each diversity index, Kruskal-Wallis test followed by the Dunn’s multiple comparisons test, ($P < 0.05$).

Supplementary Table 4: Statistical information on the semi-quantitative analysis of certain bacterial taxa

Gender	Treatment	Bacteria taxa	Samples	Kruskal-Wallis test (P value)	Dunn's multiple comparisons test	
Male	Irradiation	*Aeromonas*	3	**0.0002**	MPI vs. 1DMI	ns
					MPI vs. 4DMI	ns
					1DMI vs. 4DMI	**
Female	Irradiation	*Aeromonas*	3	**0.0005**	FPI vs. 1DFI	ns
					FPI vs. 4DFI	ns
					1DFI vs. 4DFI	**
Male	Control	*Aeromonas*	3	0.3070	MPC vs. 1DMC	ns
					MPC vs. 4DMC	ns
					1DMC vs. 4DMC	ns
Female	Control	*Aeromonas*	3	**0.0142**	FPC vs. 1DFC	**ns**
					FPC vs. 4DFC	ns
					1DFC vs. 4DFC	ns
Male	Irradiation	*Elizabethkingia*	3	**0.0048**	MPI vs. 1DMI	ns
					MPI vs. 4DMI	*
					1DMI vs. 4DMI	ns
Female	Irradiation	*Elizabethkingia*	3	**0.0002**	FPI vs. 1DFI	ns
					FPI vs. 4DFI	**
					1DFI vs. 4DFI	ns
Male	Control	*Elizabethkingia*	3	**0.0066**	MPC vs. 1DMC	ns
					MPC vs. 4DMC	*
					1DMC vs. 4DMC	ns
Female	Control	*Elizabethkingia*	3	**0.0012**	FPC vs. 1DFC	ns
					FPC vs. 4DFC	**
					1DFC vs. 4DFC	ns

Gender	Treatment	*Bacteria taxa*	Samples	Kruskal-Wallis test (P value)	Dunn's multiple comparisons test	
Male	Irradiation	*Enterococcus*	3	**0.0024**	MPI vs. 1DMI	*
					MPI vs. 4DMI	ns
					1DMI vs. 4DMI	ns
Female	Irradiation	*Enterococcus*	3	0.2771	FPI vs. 1DFI	ns
					FPI vs. 4DFI	ns
					1DFI vs. 4DFI	ns
Male	Control	*Enterococcus*	3	**0.0005**	MPC vs. 1DMC	**
					MPC vs. 4DMC	ns
					1DMC vs. 4DMC	ns
Female	Control	*Enterococcus*	3	0.0545	FPC vs. 1DFC	ns
					FPC vs. 4DFC	ns
					1DFC vs. 4DFC	ns

* indicates $P < 0.05$; ** indicates $P < 0.01$; ns indicates no significant difference.

Irradiation *vs.* Control	Two-tailed Mann-Whitney *U* test (P value)			Male vs. Female	Two-tailed Mann-Whitney *U* test (P value)		
	Aeromonas	*Elizabethkingia*	*Enterococcus*		*Aeromonas*	*Elizabethkingia*	*Enterococcus*
MPI vs. MPC	0.6571	> 0.9999	0.4857	Irradiated groups	**0.0204**	0.0593	0.0593
1DMI vs. 1DMC	**0.0286**	0.8286	0.0571	Control groups	**0.0055**	0.2876	0.3427
4DMI vs. 4DMC	0.3429	**0.0286**	0.1143				
FPI vs. FPC	0.1143	0.8286	> 0.9999				
1DFI vs. 1DFC	0.4857	0.4857	0.3429				
4DFI vs. 4DFC	> 0.9999	**0.0286**	0.1143				

Supplementary Table 5: Statistical values of Dunn-test or Kruskal-Wallis multiple comparison test with no adjustment for p-values

1- Pielou.s.evenness

	Comparison	Z	P
1	1DFC - 1DFI	-1.33929	0.180
2	1DFC - 4DFC	1.377193	0.168
3	1DFI - 4DFC	2.716481	0.007
4	1DFC - 4DFI	1.023418	0.306
5	1DFI - 4DFI	2.362707	0.018
6	4DFC - 4DFI	-0.35377	0.724
7	1DFC - FPC	-1.64252	0.100
8	1DFI - FPC	-0.30324	0.762
9	4DFC - FPC	-3.01972	0.003
10	4DFI - FPC	-2.66594	0.008
11	1DFC - FPI	-1.97103	0.049
12	1DFI - FPI	-0.63174	0.528
13	4DFC - FPI	-3.34822	0.001
14	4DFI - FPI	-2.99445	0.003
15	FPC - FPI	-0.3285	0.743
16	1DFC - 1DMC	-1.1245	0.261
17	1DFI - 1DMC	0.214792	0.830
18	4DFC - 1DMC	-2.50169	0.012
19	4DFI - 1DMC	-2.14792	0.032
20	FPC - 1DMC	0.518027	0.604
21	FPI - 1DMC	0.846531	0.397
22	1DFC - 1DMI	-0.80863	0.419
23	1DFI - 1DMI	0.530661	0.596
24	4DFC - 1DMI	-2.18582	0.029
25	4DFI - 1DMI	-1.83205	0.067
26	FPC - 1DMI	0.833897	0.404
27	FPI - 1DMI	1.162401	0.245
28	1DMC - 1DMI	0.31587	0.752
29	1DFC - 4DMC	0.871801	0.383
30	1DFI - 4DMC	2.211089	0.027
31	4DFC - 4DMC	-0.50539	0.613
32	4DFI - 4DMC	-0.15162	0.879
33	FPC - 4DMC	2.514324	0.012
34	FPI - 4DMC	2.842829	0.004
35	1DMC - 4DMC	1.996298	0.046
36	1DMI - 4DMC	1.680428	0.093
37	1DFC - 4DMI	1.225575	0.220
38	1DFI - 4DMI	2.564864	0.010
39	4DFC - 4DMI	-0.15162	0.879
40	4DFI - 4DMI	0.202157	0.840

41	FPC - 4DMI	2.868099	0.004
42	FPI - 4DMI	3.196603	0.001
43	1DMC - 4DMI	2.350072	0.019
44	1DMI - 4DMI	2.034202	0.042
45	4DMC - 4DMI	0.353774	0.724
46	1DFC - MPC	-1.81941	0.069
47	1DFI - MPC	-0.48012	0.631
48	4DFC - MPC	-3.1966	0.001
49	4DFI - MPC	-2.84283	0.004
50	FPC - MPC	-0.17689	0.860
51	FPI - MPC	0.151618	0.879
52	1DMC - MPC	-0.69491	0.487
53	1DMI - MPC	-1.01078	0.312
54	4DMC - MPC	-2.69121	0.007
55	4DMI - MPC	-3.04499	0.002
56	1DFC - MPI	-1.7057	0.088
57	1DFI - MPI	-0.36641	0.714
58	4DFC - MPI	-3.08289	0.002
59	4DFI - MPI	-2.72912	0.006
60	FPC - MPI	-0.06317	0.950
61	FPI - MPI	0.265331	0.791
62	1DMC - MPI	-0.5812	0.561
63	1DMI - MPI	-0.89707	0.370
64	4DMC - MPI	-2.5775	0.010
65	4DMI - MPI	-2.93127	0.003
66	MPC - MPI	0.113713	0.909

2- Richness

	Comparison	Z	P.unadj
1	1DFC - 1DFI	-1.71913	0.086
2	1DFC - 4DFC	-1.07445	0.283
3	1DFI - 4DFC	0.644672	0.519
4	1DFC - 4DFI	-0.37922	0.705
5	1DFI - 4DFI	1.339907	0.180
6	4DFC - 4DFI	0.695235	0.487
7	1DFC - FPC	-1.63064	0.103
8	1DFI - FPC	0.088484	0.929
9	4DFC - FPC	-0.55619	0.578
10	4DFI - FPC	-1.25142	0.211
11	1DFC - FPI	-1.97194	0.049
12	1DFI - FPI	-0.25281	0.800
13	4DFC - FPI	-0.89749	0.369
14	4DFI - FPI	-1.59272	0.111
15	FPC - FPI	-0.3413	0.733
16	1DFC - 1DMC	-2.93263	0.003
17	1DFI - 1DMC	-1.2135	0.225

18	4DFC - 1DMC	-1.85817	0.063
19	4DFI - 1DMC	-2.55341	0.011
20	FPC - 1DMC	-1.30199	0.193
21	FPI - 1DMC	-0.96069	0.337
22	1DFC - 1DMI	-1.33991	0.180
23	1DFI - 1DMI	0.379219	0.705
24	4DFC - 1DMI	-0.26545	0.791
25	4DFI - 1DMI	-0.96069	0.337
26	FPC - 1DMI	0.290735	0.771
27	FPI - 1DMI	0.632032	0.527
28	1DMC - 1DMI	1.59272	0.111
29	1DFC - 4DMC	0.176969	0.860
30	1DFI - 4DMC	1.896095	0.058
31	4DFC - 4DMC	1.251423	0.211
32	4DFI - 4DMC	0.556188	0.578
33	FPC - 4DMC	1.807611	0.071
34	FPI - 4DMC	2.148908	0.032
35	1DMC - 4DMC	3.109597	0.002
36	1DMI - 4DMC	1.516876	0.129
37	1DFC - 4DMI	0.467704	0.640
38	1DFI - 4DMI	2.18683	0.029
39	4DFC - 4DMI	1.542158	0.123
40	4DFI - 4DMI	0.846923	0.397
41	FPC - 4DMI	2.098346	0.036
42	FPI - 4DMI	2.439643	0.015
43	1DMC - 4DMI	3.400331	0.001
44	1DMI - 4DMI	1.807611	0.071
45	4DMC - 4DMI	0.290735	0.771
46	1DFC - MPC	-2.81886	0.005
47	1DFI - MPC	-1.09974	0.271
48	4DFC - MPC	-1.74441	0.081
49	4DFI - MPC	-2.43964	0.015
50	FPC - MPC	-1.18822	0.235
51	FPI - MPC	-0.84692	0.397
52	1DMC - MPC	0.113766	0.909
53	1DMI - MPC	-1.47895	0.139
54	4DMC - MPC	-2.99583	0.003
55	4DMI - MPC	-3.28657	0.001
56	1DFC - MPI	-3.46353	0.001
57	1DFI - MPI	-1.74441	0.081
58	4DFC - MPI	-2.38908	0.017
59	4DFI - MPI	-3.08432	0.002
60	FPC - MPI	-1.83289	0.067
61	FPI - MPI	-1.4916	0.136
62	1DMC - MPI	-0.53091	0.595
63	1DMI - MPI	-2.12363	0.034

64	4DMC - MPI	-3.6405	0.000
65	4DMI - MPI	-3.93124	0.000
66	MPC - MPI	-0.64467	0.519

3-Shannon

	Comparison	Z	P.unadj
1	1DFC - 1DFI	-1.19969	0.230
2	1DFC - 4DFC	1.136545	0.256
3	1DFI - 4DFC	2.336231	0.019
4	1DFC - 4DFI	0.883979	0.377
5	1DFI - 4DFI	2.083666	0.037
6	4DFC - 4DFI	-0.25257	0.801
7	1DFC - FPC	-1.61642	0.106
8	1DFI - FPC	-0.41673	0.677
9	4DFC - FPC	-2.75296	0.006
10	4DFI - FPC	-2.5004	0.012
11	1DFC - FPI	-1.86899	0.062
12	1DFI - FPI	-0.6693	0.503
13	4DFC - FPI	-3.00553	0.003
14	4DFI - FPI	-2.75296	0.006
15	FPC - FPI	-0.25257	0.801
16	1DFC - 1DMC	-1.26283	0.207
17	1DFI - 1DMC	-0.06314	0.950
18	4DFC - 1DMC	-2.39937	0.016
19	4DFI - 1DMC	-2.14681	0.032
20	FPC - 1DMC	0.353592	0.724
21	FPI - 1DMC	0.606157	0.544
22	1DFC - 1DMI	-0.73244	0.464
23	1DFI - 1DMI	0.467246	0.640
24	4DFC - 1DMI	-1.86899	0.062
25	4DFI - 1DMI	-1.61642	0.106
26	FPC - 1DMI	0.883979	0.377
27	FPI - 1DMI	1.136545	0.256
28	1DMC - 1DMI	0.530388	0.596
29	1DFC - 4DMC	1.123917	0.261
30	1DFI - 4DMC	2.323603	0.020
31	4DFC - 4DMC	-0.01263	0.990
32	4DFI - 4DMC	0.239937	0.810
33	FPC - 4DMC	2.740336	0.006
34	FPI - 4DMC	2.992902	0.003
35	1DMC - 4DMC	2.386744	0.017
36	1DMI - 4DMC	1.856357	0.063
37	1DFC - 4DMI	1.401739	0.161
38	1DFI - 4DMI	2.601425	0.009
39	4DFC - 4DMI	0.265194	0.791
40	4DFI - 4DMI	0.517759	0.605

41	FPC - 4DMI	3.018158	0.003
42	FPI - 4DMI	3.270724	0.001
43	1DMC - 4DMI	2.664567	0.008
44	1DMI - 4DMI	2.134179	0.033
45	4DMC - 4DMI	0.277822	0.781
46	1DFC - MPC	-1.81847	0.069
47	1DFI - MPC	-0.61879	0.536
48	4DFC - MPC	-2.95502	0.003
49	4DFI - MPC	-2.70245	0.007
50	FPC - MPC	-0.20205	0.840
51	FPI - MPC	0.050513	0.960
52	1DMC - MPC	-0.55564	0.578
53	1DMI - MPC	-1.08603	0.277
54	4DMC - MPC	-2.94239	0.003
55	4DMI - MPC	-3.22021	0.001
56	1DFC - MPI	-1.80584	0.071
57	1DFI - MPI	-0.60616	0.544
58	4DFC - MPI	-2.94239	0.003
59	4DFI - MPI	-2.68982	0.007
60	FPC - MPI	-0.18942	0.850
61	FPI - MPI	0.063141	0.950
62	1DMC - MPI	-0.54302	0.587
63	1DMI - MPI	-1.0734	0.283
64	4DMC - MPI	-2.92976	0.003
65	4DMI - MPI	-3.20758	0.001
66	MPC - MPI	0.012628	0.990

4- Simpson

	Comparison	Z	P.unadj
1	1DFC - 1DFI	-1.33976	0.180
2	1DFC - 4DFC	1.440876	0.150
3	1DFI - 4DFC	2.780637	0.005
4	1DFC - 4DFI	1.086976	0.277
5	1DFI - 4DFI	2.426738	0.015
6	4DFC - 4DFI	-0.3539	0.723
7	1DFC - FPC	-1.55463	0.120
8	1DFI - FPC	-0.21487	0.830
9	4DFC - FPC	-2.9955	0.003
10	4DFI - FPC	-2.64161	0.008
11	1DFC - FPI	-1.68102	0.093
12	1DFI - FPI	-0.34126	0.733
13	4DFC - FPI	-3.1219	0.002
14	4DFI - FPI	-2.768	0.006
15	FPC - FPI	-0.12639	0.899
16	1DFC - 1DMC	-1.08698	0.277
17	1DFI - 1DMC	0.252785	0.800

18	4DFC - 1DMC	-2.52785	0.011
19	4DFI - 1DMC	-2.17395	0.030
20	FPC - 1DMC	0.467653	0.640
21	FPI - 1DMC	0.594045	0.552
22	1DFC - 1DMI	-1.02378	0.306
23	1DFI - 1DMI	0.315982	0.752
24	4DFC - 1DMI	-2.46466	0.014
25	4DFI - 1DMI	-2.11076	0.035
26	FPC - 1DMI	0.530849	0.596
27	FPI - 1DMI	0.657242	0.511
28	1DMC - 1DMI	0.063196	0.950
29	1DFC - 4DMC	0.770995	0.441
30	1DFI - 4DMC	2.110757	0.035
31	4DFC - 4DMC	-0.66988	0.503
32	4DFI - 4DMC	-0.31598	0.752
33	FPC - 4DMC	2.325624	0.020
34	FPI - 4DMC	2.452017	0.014
35	1DMC - 4DMC	1.857971	0.063
36	1DMI - 4DMC	1.794775	0.073
37	1DFC - 4DMI	1.289205	0.197
38	1DFI - 4DMI	2.628966	0.009
39	4DFC - 4DMI	-0.15167	0.879
40	4DFI - 4DMI	0.202228	0.840
41	FPC - 4DMI	2.843834	0.004
42	FPI - 4DMI	2.970226	0.003
43	1DMC - 4DMI	2.376181	0.017
44	1DMI - 4DMI	2.312985	0.021
45	4DMC - 4DMI	0.51821	0.604
46	1DFC - MPC	-1.78214	0.075
47	1DFI - MPC	-0.44237	0.658
48	4DFC - MPC	-3.22301	0.001
49	4DFI - MPC	-2.86911	0.004
50	FPC - MPC	-0.22751	0.820
51	FPI - MPC	-0.10111	0.919
52	1DMC - MPC	-0.69516	0.487
53	1DMI - MPC	-0.75836	0.448
54	4DMC - MPC	-2.55313	0.011
55	4DMI - MPC	-3.07134	0.002
56	1DFC - MPI	-1.57991	0.114
57	1DFI - MPI	-0.24015	0.810
58	4DFC - MPI	-3.02078	0.003
59	4DFI - MPI	-2.66688	0.008
60	FPC - MPI	-0.02528	0.980
61	FPI - MPI	0.101114	0.919
62	1DMC - MPI	-0.49293	0.622
63	1DMI - MPI	-0.55613	0.578

64	4DMC - MPI	-2.3509	0.019
65	4DMI - MPI	-2.86911	0.004
66	MPC - MPI	0.202228	0.840
